# Genome Mining and Biological Engineering of Type III Borosins from Bacteria

**DOI:** 10.3390/ijms25179350

**Published:** 2024-08-29

**Authors:** Kuang Xu, Sijia Guo, Wei Zhang, Zixin Deng, Qi Zhang, Wei Ding

**Affiliations:** 1 State Key Laboratory of Microbial Metabolism, School of Life Sciences & Biotechnology, Shanghai Jiao Tong University, Shanghai 200240, China; score_xu@sjtu.edu.cn (K.X.); sijiag@sjtu.edu.cn (S.G.); zxdeng@sjtu.edu.cn (Z.D.); qizhang_chem@sjtu.edu.cn (Q.Z.); 2 Key Laboratory of Extreme Environmental Microbial Resources and Engineering of Gansu Province, Northwest Institute of Eco-Environment and Resources, Chinese Academy of Sciences, Lanzhou 730000, China; bestdw@163.com

**Keywords:** RiPPs, KchMA, backbone N-methylation, rational design

## Abstract

Borosins are a class of ribosomally synthesized and post-translationally modified peptides (RiPPs) with α-N-methylated backbones. Although the first mature compound of borosin was reported in 1997, the biosynthetic pathway was elucidated 20 years later. Until this work, borosins have been able to be categorized into 11 types based on the features of their protein structure and core peptides. Type III borosins were reported only in fungi initially. In order to explore the sources and potential of type III borosins, a precise genome mining work of type III borosins was conducted in bacteria and KchMA’s self-methylation activity was validated by biochemical experiment. Furthermore, a commercial protease and AI-assisted rational design was employed to engineer KchMA for the capacity to produce various N-methylated peptides. Our work demonstrates that type III borosins are abundant not only in eukaryotes but also in bacteria and have immense potential as a tool for synthetic biology.

## 1. Introduction

Methylation modification is widely prevalent in biological organisms and holds significant implications for essential life processes. The process involves the addition of a methyl group to a substrate, which can be DNA [1], RNA2 [2], proteins [3], or small molecules [4]. Methylation modification not only holds significant importance within biological systems but also finds extensive applications in the pharmaceutical industry. The introduction of methyl groups can enhance the physicochemical properties and biological activity of certain drug molecules. This modification can lead to a substantial increase in the biological activity of some compounds, a phenomenon often referred to as the “magical methyl effect” [5]. For peptide-based drugs, the backbone-N-methylation modification of the backbone significantly enhances the stability of the peptide within the biological system. Moreover, it enhances several crucial pharmacological properties, including stability, membrane permeability, and bioavailability [6]. Notably, the chemical synthesis of peptides involving backbone N-methylation typically entails numerous intricate steps and can prove to be inherently challenging [7]. Thus, identifying suitable enzymes to facilitate the backbone N-methylation of peptides through biocatalysis holds noteworthy significance in peptide drug research.

Synthesis of backbone N-methylated peptides in biological systems often occurs through non-ribosomal peptide synthetases (NRPSs), which are large multifunctional enzymes capable of synthesizing a wide variety of peptide-based natural products, such as immunosuppressants cyclosporine A and mycotoxins enniatins [8]. However, the utilization of NRPSs is limited by the difficulty of engineering such large multidomain enzymes. Recent studies have indicated that omphalotins, a class of cyclic peptides featuring up to eight backbone N-methylations, are synthesized via ribosomal processes and subsequently undergo post-translational modifications as ribosomally synthesized and post-translationally modified peptides (RiPPs) [9]. The biosynthetic gene cluster of omphalotins is very distinct from others. Its methyltransferase and the core peptide are situated at the N-terminus and C-terminus, respectively, of a singular protein known as OphMA [10,11]. Further biochemical and structural biology evidence suggests that OphMA forms a homodimer in a “head-to-tail” arrangement, and the methyltransferase domain of one monomer catalyzes the core peptide of the other [12,13]. As a result of this distinctive catalytic mechanism, this category of RiPPs has been denoted “borosins”, drawing inspiration from the mythological entity Ouroboros.

**Figure 1:**
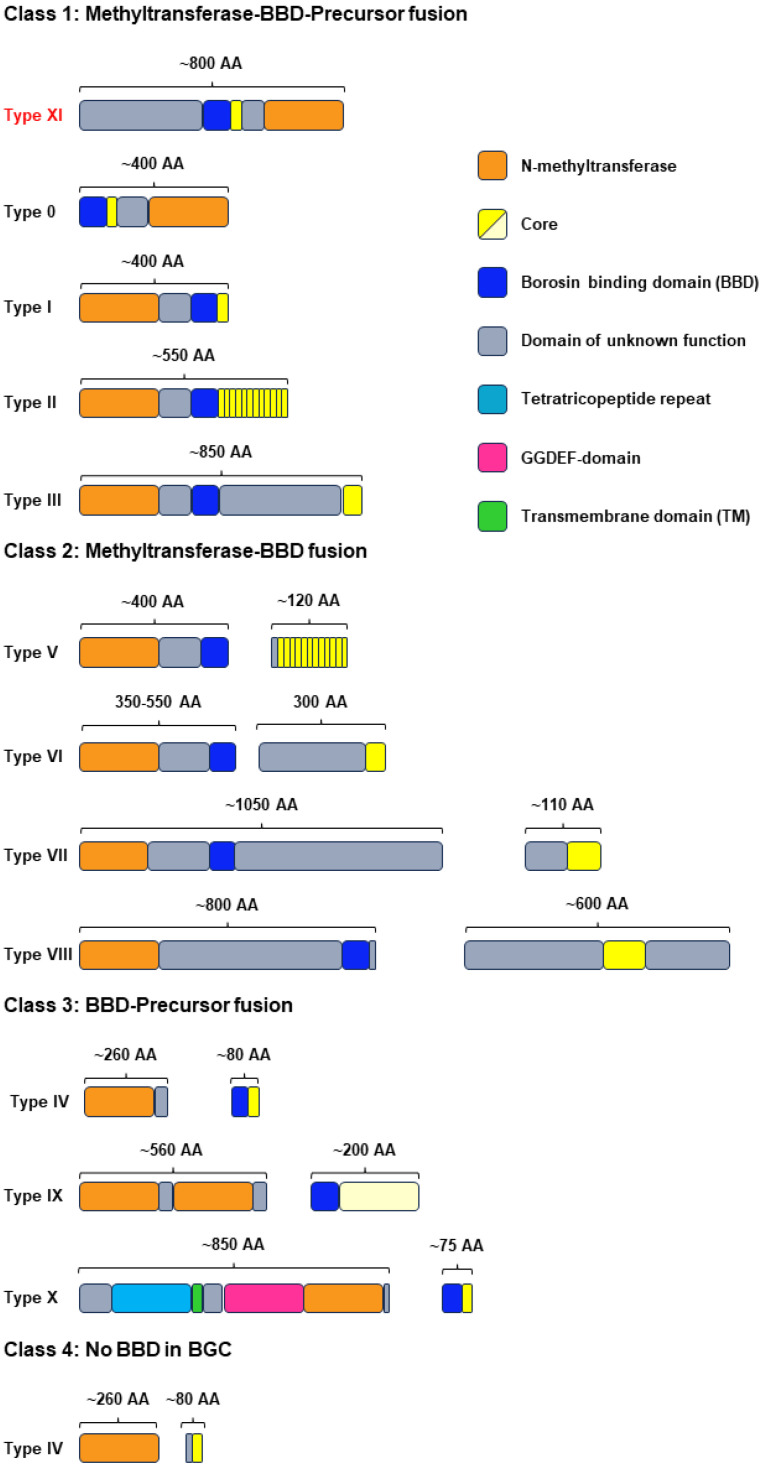
The architecture of different types of borosins. Core peptides that were not well defined were colored pale yellow. Type XI is a newly discovered borosin in this work. Class 1: the methyltransferase domain, BBD (borosin-binding domain), and core peptide are all fused within a single protein. Class 2: the methyltransferase domain and BBD are covalently linked into one protein, while the core peptide is a separate protein. Class 3: the BBD and core peptide are fused into one protein, with the methyltransferase being a distinct protein. Class 4: the methyltransferase and core peptide are individual proteins, and no BBD has been identified within their biosynthetic gene clusters. GGDEF, GGDEF-sequence-containing domain. BGC, biosynthetic gene cluster.

Bioinformatic mining work has revealed the diversity of borosins. Until now, they have been classified into 11 types (0–X) based on the protein structure and characteristics of the core peptides (Figure 1). Types I, II, and III were initially discovered in fungi [14]. In 2021, type IV borosins were uncovered from *Shewanella oneidensis*. The gene cluster of type IV differs from types I–III but is similar to traditional RiPPs, of which methyltransferase and core peptides are independent proteins [15]. Thus, type IV is reported as split borosins and types I–III are fused borosins. Like fused borosins, types V–X split borosins were found by bioinformatic prediction and biochemical experiments [16,17]. In a more recent work, Mitchell and Freenman’s group added borosins into RODEO, a genome mining tool for RiPPs. This led to the discovery of type 0 borosins, which boast core peptides at their N-terminus and methyltransferases at their C-terminus [18]. Initially, it was presumed that type II and III borosins were exclusive to the fungal kingdom. Subsequent research also postulated the existence of type II and III borosins in prokaryotic organisms. However, this hypothesis has not been empirically validated to date. In addition, the catalytic mechanisms of only a few types of borosins have been elucidated, with the remainder still not fully understood.

## 2. Results

### 2.1. Genome Mining of New Type III Borosins

**Figure 2:**
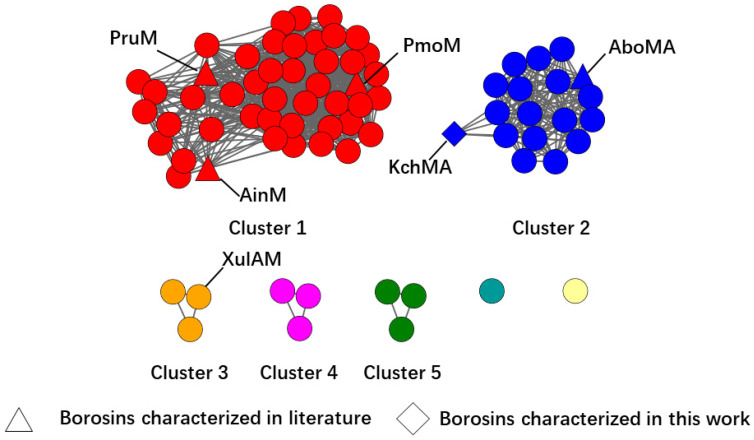
The colored sequence similarity network for mining type III borosins. Proteins with higher than 90% similarity are consolidated into one single node, and the edges connecting the nodes indicate sequence similarity. Borosins characterized in the literature are highlighted by triangles. Borosins characterized in this work are highlighted by diamonds.

Our genome mining plan targeted the discovery of type III borosins. An initial basic local alignment search tool (BLAST) analysis with typical type III borosins AboMA (P9WEN4.1) from *Anomoporia bombycina* as a probe resulted in 111 sequences from the National Center for Biotechnology Information (NCBI) database. Then, a sequence similarity network (SSN) was constructed, revealing several clusters with distinct characteristics (Figure 2 and Figure S1). Cluster 1 contained methyltransferases of type VII and VIII split borosins, predominantly bacterial with one exception from *Mucoromycota*. Further genomic analysis confirmed the borosin family membership of the outlier sequence. Cluster 2 comprised type III fused borosins, predominantly fungal with a minority of bacterial origin. The core peptide sequences of type III borosins displayed high diversity with an alternating pattern of acidic and other amino acids. Clusters 3 and 4 also presented intriguing features. Cluster 3 proteins had a methyltransferase domain at the C-terminus, aligning with the recently reported type 0 borosin. However, the proteins in this cluster contain a domain of unknown function at the N-terminus, such as XulAM (WP 130412518.1), so we classified these borosins as type XI (Figure 1). The C-terminus of the borosins in cluster 4 is very similar to type VIII borosins, meaning that there is a sequence of approximately 160 amino acids following the potential core peptides, suggesting the existence of a new type of fused borosins. Cluster 5 included a metazoan origin borosin from *Paramuricea clavata*, indicating the potential for secondary metabolites in animals (Figure S1).

### 2.2. Validation of the New Type III Borosin KchMA Derived from Bacteria

**Figure 3:**
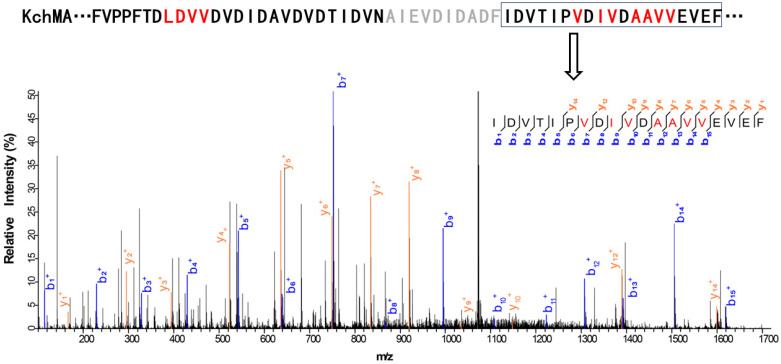
The methylation pattern of KchMA. An example of LC-MS/MS data and relative abundance for KchMA. The fragmentation patterns of the methylated peptide, as elucidated by LC-MS/MS, are also depicted. Methylated amino acids confirmed by MS/MS are marked with red fonts. The sequences that LC-MS/MS does not cover are gray fonts. Further information can be found in Figure S2 and the Supplementary Data.

We selected KchMA (WP_084224105.1) as the representation of type III in bacteria (Figure 2). To examine whether it can offer self-methylation activity, we synthesized the genes with codon optimization. Then, this gene was heterologously expressed in the *E. coli BL21 star* (DE3) strain. Due to the challenge of hydrolysis of such a long putative core peptide, we employed two proteases, chymotrypsin and elastase, to hydrolyze this protein, respectively. By combining the results from both, we found that there could be up to 11 modifications on the residues from position 742 to 796 (Figure 3, Figure S2, Supplementary Data). The MS/MS results indicated that while KchMA prefers to catalyze nonpolar amino acids, such as alanine, valine, and leucine, some modification exists on acidic amino acids, such as aspartic acid. This characteristic differs from the known type III borosin, AboMA, which can only modify non-charged amino acids. This is consistent with the SSN where KchMA is relatively far from cluster 2.

### 2.3. Introduction of Artificial Protease Sit to Release N-Methylation Peptide

**Figure 4:**
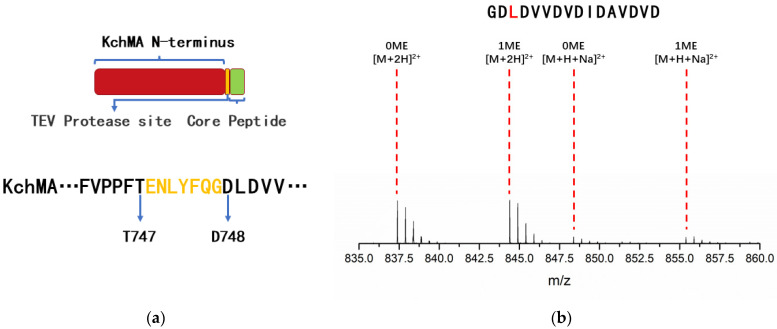
(**a**) Protein domain architecture and sequence of KchMA_TEV_. The N-terminus of KchMA, TEV cleavage site, and the core peptide are represented by red, yellow, and green rectangles, respectively. The TEV cleavage site is inserted between the wild-type KchMA T747 and D748 and is highlighted in yellow. (**b**) The methylation result of KchMA_TEV_. The methylated amino acids confirmed by MS/MS are marked with red fonts. Detailed information for each ion (*m*/*z*, charge state, and deviation) can be found in the Supplementary Data.

Despite the widespread distribution of borosin gene clusters in nature, few mature compounds have been identified to date due to the absence of a specific peptidase. However, some commercial proteases can be used to release core peptides from large fused borosins. This approach requires introducing a recognition site in front of the core peptide. The tobacco etch virus (TEV) protease has a well-defined specificity for cleaving peptide bonds and high activity under variable conditions (e.g., temperature, pH, buffer composition). According to our research results above, the methyltransferase domain of KchMA has substrate promiscuity. Thus, the KchMA methyltransferase domain, a protease cleavage site, and variable core peptides combined together could biosynthesize diverse N-methylation peptides (Figure 4a). To test this design, a TEV protease cleavage site (Glu-Asn-Leu-Tyr-Phe-Gln-Gly) was inserted between T747 and D748 of KchMA to generate KchMA_TEV_. This engineered protein was cultured in the same condition as the wild type. The MS/MS results showed that only leucine at the 3-site (L3) of the TEV cleavage core peptide was methylation (Figure 4b, Figure S3a, Supplementary Data). This is distinct from wild-type KchMA with 11 site self-methylations. The incorporation of the TEV cleavage site causes alterations in the sequence of the leader peptide, thereby affecting the occurrence of modifications.

**Figure 5:**
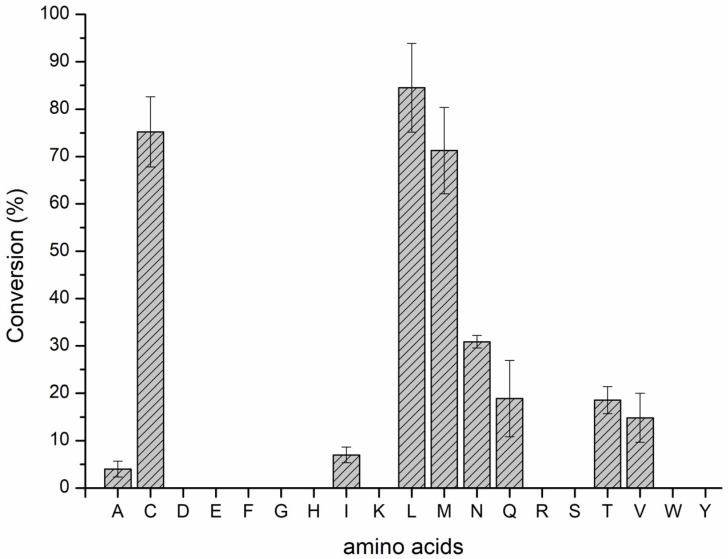
The catalytic activity of KchMA_TEV_’s methyltransferase domain for different 3-site mutant core peptides. The relative abundance was determined by integrating the EIC peaks and calculating the ratio between the integration of a one-methylation product and the sum of the zero and one-methylation core peptides. Each experiment was conducted in triplicate. The label on the ox-axis represents the single-letter abbreviations for amino acids. Amino acids without corresponding bars on the graph indicated that methylation modifications were not detected.

We reasonably speculated that KchMA_TEV_’s methyltransferase specifically modified the third amino acid after the TEV cut site. Therefore, changing the L3 residue of KchMA_TEV_ will generate a series of 3-site N-methylation peptides. We mutated the L3 to other natural amino acids except proline. The results showed that KchMA_TEV_’s methyltransferase preferred modifying amino acids with aliphatic side chains but had the highest modification efficiency towards leucine residues. For polar amino acids, cystine is mostly modified, and the conversion comes to over 75%. However, the acid and basic amino acids, as well as the aromatic amino acids, cannot be methylated (Figure 5, Figure S3, Supplementary Data). Although the catalytic selectivity of methyltransferases remained mysterious and should be determined by combining the position and chemical properties of substrate residues, this biological engineering is proven to potentially produce various N-methylation peptides.

### 2.4. Rational Evolution of Methyltransferase Domain of KchMA_TEV_

To further improve the efficiency of the methyltransferase of KchMA_TEV_, we tried to evolve the methyltransferase by AI-assisted protein engineering. First, the identification of key residues was carried out. By comparing with OphMA, residues Y73, R79, and Y83 of KchMA_TEV_ may be related to catalytic activity; Y105 and S136 may be crucial to SAM binding; and residues Y70 and Q179 may bind to substrate (Figure S4). We constructed Y70A, Y73F, R79A, Y83F, Y105A, S136A, and Q179A mutants. Compared to KchMA_TEV_, Y70A exhibited a moderate decrease in activity, while mutants R79A, Y83F, and Q179A showed a significant reduction in activity. Y105A and S136A drastically decreased, and the modified peptides were virtually undetectable (Figure S5). To our surprise, Y73F mutant retained most of the activity, which is inconsistent with the OphMA and dbOphMA results. To further discuss the role of Y73, we mutated this site to alanine. The Y73A mutants showed lower activity towards substrate peptides (Figure S5).

Then, we built a crystal structure model of the methyltransferase domain of KchMA_TEV_ and substrate, as well as SAM (Figure S6). There are nine residues close to L3 of the core peptide within 5 Å left after excluding the key residues for catalysis. The conserved residues were filtered out by multiple sequence alignment using Clustal Omega [19] (Figure S4). We conducted an alanine-scanning experiment on these sites to determine the critical residues for activity. Among them, the C49A and F188A mutants exhibited significant changes compared to the wild type, of which C49A showed the most substantial decrease in activity (Figure S7).

**Figure 6:**
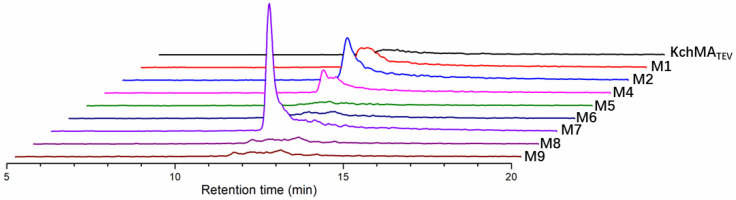
The extracted ion chromatogram (EIC) of two-methylation product of each mutant. The EIC peak for *m*/*z* 1701.8065 [M + H]^+^ revealed accumulation of the peptides with two backbone-N-methylations.

Model action results guided us to select C49 and F188 for further enzyme engineering. Then, we built an enzyme library with the AI tool FuncLib, which offers an automated approach to designing enzyme active-site mutants, significantly reducing the labor and time-consuming experiments required by traditional methods [20]. In the computational process of FuncLib, the conformations of key residues mentioned above were preserved. Based on phylogenetic analysis and Rosetta design calculations, FuncLib provided nine mutants (M1–M9) that included one or two mutations each (Table 1). Among them, M3 was excluded because its C49A mutation had proven deleterious to activity in previous studies. Then, the remaining eight mutants were conducted. It was surprising that variants M1 and M2 exhibited dimethylation activity, and M7 showed the most significant dimethylation efficiency (Figure 6). MS/MS results confirmed that the second methylated site was on A12 (Figure S8, Supplementary Data).

## 3. Discussion

The results of our study significantly expand the taxonomic distribution of borosins. The SSN analysis of type III borosins revealed a complex landscape of sequence diversity and evolutionary relationships. The identification of split borosins in fungi and the presence of a new type of fused borosins in metazoans highlight the potential for novel biological functions and metabolic pathways. The methyltransferase domain’s positioning at the C-terminus in cluster 3 of Figure 1 indicates a broader distribution than initially recognized and warrants further investigation.

**Figure 7:**
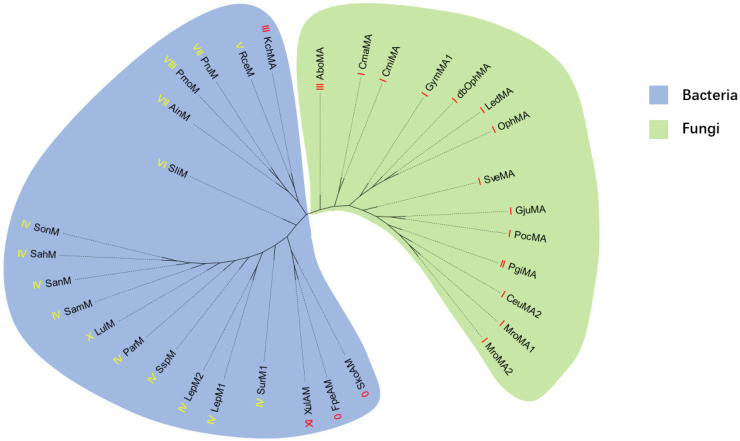
Phylogenetic tree of representative borosins. The type of each borosin is indicated in front of its name, with the split borosins marked in yellow and the fused borosins marked in red. The background color of the phylogenetic tree clades represents the source of the proteins, with those from fungi colored blue and those from bacteria colored green.

To further elucidate the evolutionary relationships of borosins, a phylogenetic tree was constructed (Figure 7). The methodological approach involved using full-length sequences for fused borosins and methyltransferase for split borosins [14,16,17,18], employing the maximum likelihood method for tree construction. Notably, the phylogenetic distribution of borosins does not conform to the previously established classifications [18]. Instead, its distribution patterns correspond to the distribution of taxonomy. Fungal-derived borosins are distinctly clustered on one clade, while their bacterial counterparts are found on the other. This clear demarcation suggests a divergence in evolutionary paths between these two groups. KchMA, a type III borosin, exhibits closer affinity to RceM AinM, PruM, and PmoM, which are type V, VII, and VIII borosins [16,17], compared to AboMA [14]. The clustering of XulAM, FpeAM, and SkoAM, which possess a methyltransferase domain at the C-terminus [18], indicates a shared evolutionary trait among these proteins. Furthermore, the close relationship between types III, VII, and VIII on the phylogenetic tree and their placement at the interface between fungi and bacteria may indicate ancient evolutionary processes. Events such as endosymbiosis, horizontal gene transfer, or periods of extensive genetic exchange could have contributed to this distribution pattern [21]. The phylogenetic positioning of these borosin types at the boundary of fungi and bacteria could also reflect their role in early divergence or a common ancestry shared by these organisms [18]. Understanding these evolutionary relationships is pivotal for appreciating the biological significance of borosins. It helps the rational engineering of these proteins through approaches such as ancestral sequence reconstruction. As genomic data accumulate, the resolution of these phylogenetic relationships will improve, potentially revealing more about the evolutionary history and functional diversification of borosins. Future research should focus on integrating additional genomic data to refine the phylogenetic tree and further explore the complex evolutionary dynamics of borosins.

The results of our bioengineering study highlight the potential for precise enzymatic cleavage and modification of borosins, expanding the scope of peptide engineering. The use of TEV protease for the cleavage of engineered borosins introduces a strategy that could be broadly applied to other peptide systems. Except for the strategy applied in this work, the intein-mediated protein ligation (EPL) strategy [22] and bioconjugation strategy [23] were reported by Song et al. and Zheng et al., which expanded the scope of substrates. However, the requirement for intein fusion and chemical ligation steps adds complexity to the overall process and may introduce additional variability in terms of ligation efficiency. Furthermore, the use of inteins and small molecules may be limited by their stability and activity under the desired reaction conditions, which could affect the yield and quality of the final product.

We have found the engineered protein KchMA_TEV_ furthers the unique capacity to catalyze the leucine residue at the core peptide’s third position. Substrate promiscuity experiments have revealed that the methyltransferase’s site selectivity remains unaffected even when the amino acid at the third position is substituted with various other residues. This discovery emphasizes the exceptional specificity of KchMA_TEV_ for its target site, rendering it highly advantageous for the biosynthesis of 3-N-methylation peptides.

The KchMA_TEV_ demonstrates a proclivity for catalyzing nonpolar amino acids with aliphatic side chains in the core peptide. This inclination indicates a potential correlation with the unique attributes of KchMA_TEV_’s methyltransferase active-site pocket. Scrutiny of the amino acid residues surrounding the active center, particularly phenylalanine at positions 188 and 193, reveals the prevalence of sizable, aromatic amino acids with hydrophobic side chains. These residues contribute to establishing a relatively hydrophobic milieu within the active site. The hydrophobic nature of the active site, accentuated by substantial aromatic side chains, appears to exert a pivotal influence on KchMA_TEV_’s substrate selectivity. It engenders a microenvironment particularly conducive to the binding and catalysis of nonpolar, aliphatic amino acids. It also concurrently imposes steric constraints that challenge the accommodation of amino acids with larger or more intricate side chains [24].

To enhance the methyltransferase activity in KchMA_TEV_, we employed bioinformatics techniques alongside site-directed mutation to pinpoint the critical residues within the methyltransferase domain. Our focused investigation centered on the function of Y73, which revealed notably heightened activity in Y73F mutants compared to Y73A mutants. Given that the phenyl ring on the amino acid side chain at the corresponding position of wild-type PgiMA is phenylalanine [14], we inferred its pivotal role in shaping the active site and posited that the presence of hydroxyl groups may augment catalytic efficacy. Furthermore, our observations demonstrated that mutations C49A and F188A caused a reduction in enzymatic activity, signifying the influence of additional sites within the active pocket on catalytic function alongside the well-documented key residues reported in the literature.

The utilization of FuncLib presents a promising approach for the rapid generation of borosin variants with favorable activities. FuncLib necessitates the input of mutation sites. At each site, the sequence space is restricted through evolutionary conservation analysis (position-specific scoring matrix [PSSM]) and mutational scanning stability calculations (ΔΔG). Subsequently, potential multi-point mutants are enumerated utilizing Rosetta’s atomic design calculations. Following this, the designs are prioritized based on energy, and the sequences are clustered to form a repertoire for experimental evaluation [21]. This methodology has effectively reduced experimental time and surmounted the challenges associated with high-throughput screening. A variant, M7, capable of methylating L3 and A12 of core peptides has been successfully obtained. Although the efficiency of dimethylation warrants improvement, this discovery unveils new prospects for the targeted modification of peptides and indicates that further refinement could yield enzymes with novel specificities.

However, further evidence from structural biology and molecular dynamics studies is required to fully elucidate the mechanisms underlying the site selectivity and substrate preferences of KchMA_TEV_ and its variants. These advanced techniques can provide insights into the protein’s behavior and interactions at the molecular level, which are essential for understanding the enzyme’s catalytic properties [15]. By capturing and simulating the conformations of KchMA_TEV_ at various time points, we can gain a detailed picture of how the enzyme’s active site engages in the methylation of multiple sites on the core peptide. This dynamic view of the enzyme’s action could reveal the structural transitions and interactions that facilitate its substrate specificity and catalytic efficiency. Understanding the substrate preferences and site-selectivity mechanisms can guide the engineering of KchMA_TEV_ to further improve its catalytic efficiency, alter its substrate range, or enhance its stability under various conditions.

The future application of the KchMA_TEV_ involves its integration with other synthetic biology tools, such as codon expansion and phage display, which presents a highly promising avenue of research. Codon expansion [25] offers the potential to introduce non-natural amino acids (NCAAs) to the core peptides, enabling the fermentation-based production of peptides with both backbone N-methylations and NCAAs. Furthermore, phage display [26] can be employed to construct a library of peptides with backbone N-methylations, followed by high-throughput screening to isolate peptide drugs with diverse biological activities.

## 4. Materials and Methods

### 4.1. Materials

Tryptone and yeast extract were purchased from Oxoid Ltd., London, UK. Other reagents for medium and buffer were purchased from Sinopharm Chemical Reagent Co., Ltd., Shanghai, China. The antibiotics used for molecular biology experiments were purchased from Shanghai Titan Technology Co., Ltd., Shanghai, China. The reagents for LC-MS were purchased from Anpel Laboratory Technologies Inc., Shanghai, China.

Gene synthesis and codon optimization were performed by Sangon Biotech (Shanghai) Co., Ltd. (Shanghai, China). Primers used in this work were synthesized by Beijing Tsingke Biotech Co., Ltd. (Beijing, China) and listed in Table S1. Phanta Max Super-Fidelity DNA polymerase and Clonexpress II One-Step Cloning Kit was purchased from Nanjing Vazyme Biotech Co., Ltd. (Nanjing, China). The restriction enzymes were purchased from Thermo Fisher Scientific Co., Ltd. (Waltham, MA, USA).

### 4.2. Genome Mining

Protein sequences of AboMA were subjected to BLAST searches, with proteins of the requisite length identified and selected accordingly. For type III borosins, a sequences similarity network was built by EFI-EST [27,28]. The alignment score was set as 130. The network was visualized and edited using Cytoscape software 3.10.2.

### 4.3. Construction of Phylogenetic Tree

The Clustal Omega online tool [19] was utilized for the multiple sequence alignment, with the results being submitted to IQ-TREE [29] for the construction of the phylogenetic tree. Subsequently, the tree was visualized and edited using the Chiplot online tool [30].

### 4.4. AI-Assisted Protein Engineering

The model of the methyltransferase domain with substrate peptide and SAH was built by the Swiss model [31] based on the structure of dbOphMA (PDB: 6MJG). The structure of KchMA_TEV_ was predicted by Rosetta fold using the Rosetta online server [32]. The first result of the Rosetta fold prediction was submitted to the FuncLib online server [20]. The key residues were fixed during the computation.

### 4.5. Construction of Mutants

Primers for mutagenesis are listed in Table S1. A TEV cleavage site was introduced after T746 of KchMA using the plasmid encoding the wild-type KchMA as a PCR template. The expression plasmids encoding the mutants of KchMA_TEV_ were constructed by inverse PCR mutagenesis [33]. Plasmids were sequenced using T7 and T7-terminator primers.

### 4.6. Protein Expression

The process of protein expression is consistent with the previous literature reports with several modifications [10]. Briefly, the recombinant BL21 star (DE3) strain was inoculated into Luria-Bertani (LB) medium (0.5% (*w*/*v*) yeast extract, 1% (*w*/*v*) tryptone, 1% (*w*/*v*) sodium chloride) containing 50 μg/mL kanamycin and incubated overnight at 37 °C. Then, the culture was diluted 1:100 into terrific broth (TB) medium (2.4% (*w*/*v*) yeast extract, 1.2% (*w*/*v*) tryptone, 0.4% (*w*/*v*) glycerol, 0.017 M KH_2_PO_4_ and 0.072 M K_2_HPO_4_) with 50 μg/mL kanamycin and grown at 37 °C until the OD_600_ reached 1.5–2. After cooling on ice for 30 min, isopropyl-β-D-thiogalactopyranoside (IPTG) was added to a final concentration of 0.2 mM, and the culture was incubated at 16 °C for five days.

### 4.7. Protein Purification

The process of protein purification is consistent with the previous literature reports with several modifications [34]. Briefly, the bacterial culture was centrifuged at 4000 rpm for ten minutes to pellet the cells with a high-speed centrifuge (Beckman Coulter, Inc., Chaska, MN, USA, Avanti JXN-26). The supernatant was then decanted, and the cell pellet was resuspended in lysis buffer (40 mM tris, 200 mM NaCl, 10 mM imidazole, 10% glycerol, pH = 8.0). Subsequently, a pressure homogenizer (Shanghai Litu Ultra-High-Pressure Equipment Co., Ltd., Shanghai, China, fb-110x15-PLUS) was used to lyse the bacteria. After lysis, the lysed bacterial solution was centrifuged at 15,000 rpm for 30 min to separate the soluble proteins from the cell debris and insoluble material. The supernatant, which contains the soluble proteins, was then incubated with nickel affinity resin at 4 °C for 1 h. Wash buffer (40 mM tris, 200 mM NaCl, 50 mM imidazole, 10% glycerol, pH = 8.0) was used to remove any contaminant proteins from the column, while elution buffer (40 mM tris, 200 mM NaCl, 500 mM imidazole, 10% glycerol, pH = 8.0) was employed to dissociate the target protein from the resin. Ultrafiltration was utilized to concentrate the protein and exchange the buffer, resulting in a protein solution ready for subsequent hydrolysis reactions.

### 4.8. The Enzymatic Hydrolysis of Borosins

Chymotrypsin hydrolysis was performed at a substrate-to-enzyme ratio of 50:1, with the reaction mixture supplemented with calcium ions at a final concentration of 5 mM; the reaction is carried out at 30 °C for 12 h. Elastase hydrolysis was conducted at a substrate-to-enzyme ratio of 20:1, with the reaction proceeding at 30 °C for 4 h. TEV protease hydrolysis followed a substrate-to-enzyme ratio of 20:1, and the reaction was incubated at 4 °C for 12 h. Upon completion of the reactions, an equal volume of acetonitrile or 1% was added to quench the reactions.

### 4.9. LC-MS/MS Analysis

Mobile phase A was ultra-pure water containing formic acid at a concentration of 0.1% (*v*/*v*), while mobile phase B was acetonitrile also containing formic acid at a concentration of 0.1% (*v*/*v*). Data were recorded on a Agilent Q-TOF 6546 mass spectrometer equipped with a 1290 Infinity II LC system (Agilent Technologies, Inc., Santa Clara, CA, USA) using a Thermo Scientific Hypersil C8 column or a Agilent ZORBAX Eclipse Plus C18 column. Data were processed using Agilent MassHunter 10.0 and pLabel 2.4 software [35]. Ions with a deviation within 10 ppm were considered reliable.

## 5. Conclusions

In conclusion, our study entailed a precise genome analysis aimed at identifying type III borosins in bacteria. Utilizing bioinformatics tools such as BLAST and SSN, we sought to uncover novel type III borosins. Moreover, our investigation indicated the widespread presence of borosins, even in more advanced eukaryotes. We performed biochemical experiments to characterize the function of type III borosin KchMA originating from bacteria, marking the first such characterization. A TEV protease site was incorporated in front of the core peptide of KchMA to generate the new protein KchMA_TEV_ in order to release the core peptide with commercial protease. Additionally, residues C49, Y70, Y73, R79, Y83, Y105, S136, Q179, and F188 of KchMA_TEV_’s methyltransferase were determined to be pivotal for catalytic activity. Furthermore, we used AI tools to redesign the methyltransferase, resulting in the generation of an improved activity variant, M7. The engineer succeeded in biosynthesizing diversity peptides with backbone N-methylations, indicating the potential of borosins as essential tools in synthetic biology.

## Figures and Tables

**Table 1:** FuncLib designs based on KchMA_TEV_.

Mutants Name	Position 49	Position 188
KchMA_TEV_	C	F
M1	V	Y
M2	T	F
M3	A	W
M4	M	M
M5	S	V
M6	C	L
M7	L	H
M8	G	T
M9	I	I

## Data Availability

The data presented in this study are available in the article and Supplementary Materials.

## Supplementary Materials

The following supporting information can be downloaded at https://www.mdpi.com/article/10.3390/ijms25179350/s1.
