# Supplementary material for: Genome Mining and Biological Engineering of Type III Borosins from Bacteria
Source: Int J Mol Sci. 2024 Aug 29;25(17):9350. doi: 10.3390/ijms25179350 (PMC11395268; doi:10.3390/ijms25179350)
Supplement: Supplementary file 1 [file ijms-25-09350-s001.zip › Supplementary material.pdf]

International Journal of
Molecular Sciences

MDPI

Supplementary material

Genome Mining and Biological Engineering of Type III Borosins from Bacteria

Kuang Xu [1], Sijia Guo [1], Wei Zhang [2], Zixin Deng [1], Qi Zhang [1] and Wei Ding [1,*]

[1] State Key Laboratory of Microbial Metabolism, School of Life Sciences & Biotechnology, Shanghai Jiao Tong University, Shanghai 200240, China; score_xu@sjtu.edu.cn (K.X.); sijiag@sjtu.edu.cn (S.G.); zxdeng@sjtu.edu.cn (Z.D.); qizhang_chem@sjtu.edu.cn (Q.Z.)

[2] Key Laboratory of Extreme Environmental Microbial Resources and Engineering of Gansu Province, Northwest Institute of Eco-Environment and Resources, Chinese Academy of Sciences, Lanzhou 730000, China; bestdw@163.com

* Correspondence: weiding@sjtu.edu.cn

Table S1. Primers in this work[1].

Primers	Sequences
KT-For	CCGCGCGGCAGCCATATGACCGATGGT
KT-Rev	TCCTTGGAAGTATAGATTTTCGGTGAACGGCGGAACGAATTTCAG
TP-For	CTATACTTCCAAGGAGATTTAGATGTTGTTGATGTG
TP-Rev	GTGGTGCTCGAGTTAATCAACATCAACGGCATCG
KchMATEV-L3A-For	GGAGATgcAGATGTTGTTGATGTGGATATCGATGCC
KchMATEV-L3A-Rev	CCACATCAACAACATCTgcATCTCCTTGGAAGTATA
KchMATEV-L3C-For	GGAGATTgcGATGTTGTTGATGTGGATATCGATGCC
KchMATEV-L3C-Rev	CCACATCAACAACATCgcAATCTCCTTGGAAGTATA
KchMATEV-L3D-For	GGAGATgacGATGTTGTTGATGTGGATATCGATGCC
KchMATEV-L3D-Rev	CCACATCAACAACATCgtcATCTCCTTGGAAGTATA
KchMATEV-L3E-For	GGAGATgaAGATGTTGTTGATGTGGATATCGATGCC
KchMATEV-L3E-Rev	CCACATCAACAACATCttcATCTCCTTGGAAGTATA
KchMATEV-L3F-For	GGAGATTTtGATGTTGTTGATGTGGATATCGATGCC
KchMATEV-L3F-Rev	CCACATCAACAACATCaAAATCTCCTTGGAAGTATA
KchMATEV-L3G-For	GGAGATggcGATGTTGTTGATGTGGATATCGATGCC
KchMATEV-L3G-Rev	CCACATCAACAACATCgccATCTCCTTGGAAGTATA
KchMATEV-L3H-For	GGAGATcacGATGTTGTTGATGTGGATATCGATGCC
KchMATEV-L3H-Rev	CCACATCAACAACATCgtgATCTCCTTGGAAGTATA
KchMATEV-L3I-For	GGAGATaTAGATGTTGTTGATGTGGATATCGATGCC
KchMATEV-L3I-Rev	CCACATCAACAACATCTAtATCTCCTTGGAAGTATA
KchMATEV-L3K-For	GGAGATaaAGATGTTGTTGATGTGGATATCGATGCC
KchMATEV-L3K-Rev	CCACATCAACAACATCtttATCTCCTTGGAAGTATA
KchMATEV-L3M-For	GGAGATatgGATGTTGTTGATGTGGATATCGATGCC
KchMATEV-L3M-Rev	CCACATCAACAACATCcatATCTCCTTGGAAGTATA
KchMATEV-L3N-For	GGAGATaacGATGTTGTTGATGTGGATATCGATGCC
KchMATEV-L3N-Rev	CCACATCAACAACATCgttATCTCCTTGGAAGTATA
KchMATEV-L3Q-For	GGAGATcaAGATGTTGTTGATGTGGATATCGATGCC
KchMATEV-L3Q-Rev	CCACATCAACAACATCttgATCTCCTTGGAAGTATA
KchMATEV-L3R-For	GGAGATagAGATGTTGTTGATGTGGATATCGATGCC
KchMATEV-L3R-Rev	CCACATCAACAACATCtctATCTCCTTGGAAGTATA
KchMATEV-L3S-For	GGAGATagcGATGTTGTTGATGTGGATATCGATGCC
KchMATEV-L3S-Rev	CCACATCAACAACATCgctATCTCCTTGGAAGTATA
KchMATEV-L3T-For	GGAGATaccGATGTTGTTGATGTGGATATCGATGCC
KchMATEV-L3T-Rev	CCACATCAACAACATCggtATCTCCTTGGAAGTATA
KchMATEV-L3V-For	GGAGATgTAGATGTTGTTGATGTGGATATCGATGCC
KchMATEV-L3V-Rev	CCACATCAACAACATCTAcATCTCCTTGGAAGTATA
KchMATEV-L3W-For	GGAGATTggGATGTTGTTGATGTGGATATCGATGCC
KchMATEV-L3W-Rev	CCACATCAACAACATCccAATCTCCTTGGAAGTATA
KchMATEV-L3Y-For	GGAGATTacGATGTTGTTGATGTGGATATCGATGCC
KchMATEV-L3Y-Rev	CCACATCAACAACATCgtAATCTCCTTGGAAGTATA
Y70A-For	GCGGTTGATCTGgcTACCCTGTACGACAACGACAAACCG
Y70A-Rev	GTCGTACAGGGTAgcCAGATCAACCGCATCCGGCTGCTG
Y73A-For	GATCTGTATACCCTGgcCGACAACGACAAACCGCGTCGC
Y73A-Rev	ACGCGGTTTGTCGTTGTCGgcCAGGGTATACAGATCAAC
Y73F-For	GATCTGTATACCCTGTtCGACAACGACAAACCGCGTCGC
Y73F-Rev	ACGCGGTTTGTCGTTGTCGaACAGGGTATACAGATCAAC
R79A-For	CGACAAACCGgcTCGCATTACCTACGTCCA
R79A-Rev	TGGACGTAGGTAATGCGAgcCGGTTTGTCG
Y83F-For	CGCATTACCTtCGTCCAGATGGCTGAAAGC
Y83F-Rev	GCTTTCAGCCATCTGGACGaAGGTAATGCG

Continued Table S1. Primers in this work[1].

Primers	Sequences
Y105A-For	AATGTTGTTGGTGTGTTCgcTGGTCATCCGGGCGTTTTC
Y105A-Rev	AACGCCCGGATGACCAgcGAACACACCAACAACATTCAG
S136A-For	CTGCCGGCAGTTgCTGCGCTTGATTGCCTG
S136A-Rev	GCAATCAAGCGCAGcAACTGCCGGCAGCAT
Q179A-For	CACGTGGTACTGCTGgcGGTAGGTAGCGTTGGC
Q179A-Rev	AACGCTACCTACCgcCAGCAGTACCACGTGGCT
C49A-For	CGTGTGTTCTACgcCGTGAGCGATCCGACCACCGAACGT
C49A-Rev	CGGATCGCTCACGgcGTAGAACACACGATCCGCCCAACG
N113A-For	TTTTCGTTgcCCCGTCCCACCGTGCCATCGCT
N113A-Rev	GGACGGGgcAACGAAAACGCCCGGATGACCAT
S182A-For	TGCAGGTAGGTgcCGTTGGCGACACCGGTTTCC
S182A-Rev	AACGgcACCTACCTGCAGCAGTACCACGTGGC
F188A-For	TgcCCGCTTCGCTGGTTTCCCGAACACCCATC
F188A-Rev	AACCAGCGAAGCGGgcACCGGTGTCGCCAACGCT
F193A-For	TTCGCTGGTgcCCCGAACACCCATCTGGGCGC
F193A-Rev	TTCGGGgcACCAGCGAAGCGGAAACCGGTGTC
C49V-For	TGTGTTCTACgtCGTGAGCGATCCGACCACCGA
C49V-Rev	CTCACGacGTAGAACACACGATCCGCCCAACGC
F188Y-For	TTaCCGCTTCGCTGGTTTCCCGAACACCCATC
F188Y-Rev	AACCAGCGAAGCGGtAACCGGTGTCGCCAACGC
C49T-For	TGTGTTCTACacCGTGAGCGATCCGACCACCGA
C49T-Rev	CTCACGgtGTAGAACACACGATCCGCCCAACGC
C49M-For	GTTCTACatgGTGAGCGATCCGACCACCGAAC
C49M-Rev	CGCTCACcatGTAGAACACACGATCCGCCCAA
F188M-For	TatgCGCTTCGCTGGTTTCCCGAACACCCATC
F188M-Rev	AACCAGCGAAGCGcatACCGGTGTCGCCAACGCT
C49S-For	TGTGTTCTACagCGTGAGCGATCCGACCACCGA
C49S-Rev	CTCACGctGTAGAACACACGATCCGCCCAACGC
F188V-For	TgTCCGCTTCGCTGGTTTCCCGAACACCCATC
F188V-Rev	AACCAGCGAAGCGGAcACCGGTGTCGCCAACGCT
F188L-For	TcTCCGCTTCGCTGGTTTCCCGAACACCCATC
F188L-Rev	AACCAGCGAAGCGGAgACCGGTGTCGCCAACGCT
C49L-For	TGTGTTCTACctCGTGAGCGATCCGACCACCGA
C49L-Rev	CTCACGagGTAGAACACACGATCCGCCCAACGC
F188H-For	TcaCCGCTTCGCTGGTTTCCCGAACACCCATCT
F188H-Rev	AAACCAGCGAAGCGGtgACCGGTGTCGCCAACGCT
C49G-For	TGTGTTCTACgGCGTGAGCGATCCGACCACCG
C49G-Rev	TCACGCcGTAGAACACACGATCCGCCCAACGC
F188T-For	TacCCGCTTCGCTGGTTTCCCGAACACCCATCT
F188T-Rev	AAACCAGCGAAGCGGgtACCGGTGTCGCCAACGCT
C49I-For	TGTTCTACatCGTGAGCGATCCGACCACCGAAC
C49I-Rev	CGCTCACGatGTAGAACACACGATCCGCCCAAC
F188I-For	TaTCCGCTTCGCTGGTTTCCCGAACACCCATC
F188I-Rev	AACCAGCGAAGCGGAtACCGGTGTCGCCAACGCT

[1] The restriction sites are shown underlined, and the mutation sites are shown in lowercase letters.

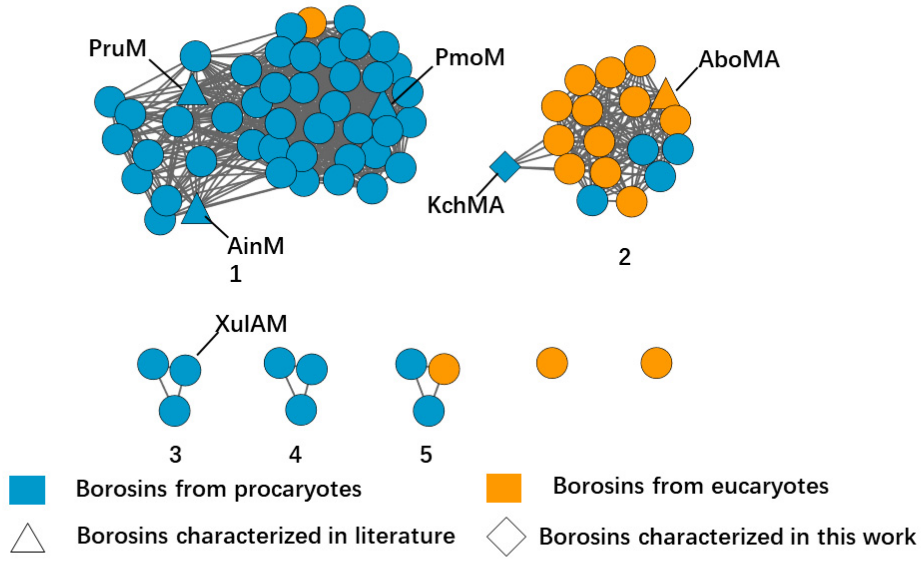

Figure S1. The sequence similarity network for mining type III and type XI borosins. The image layout corresponds with Figure 1 in the main text but is colored according to the source species.

(a)

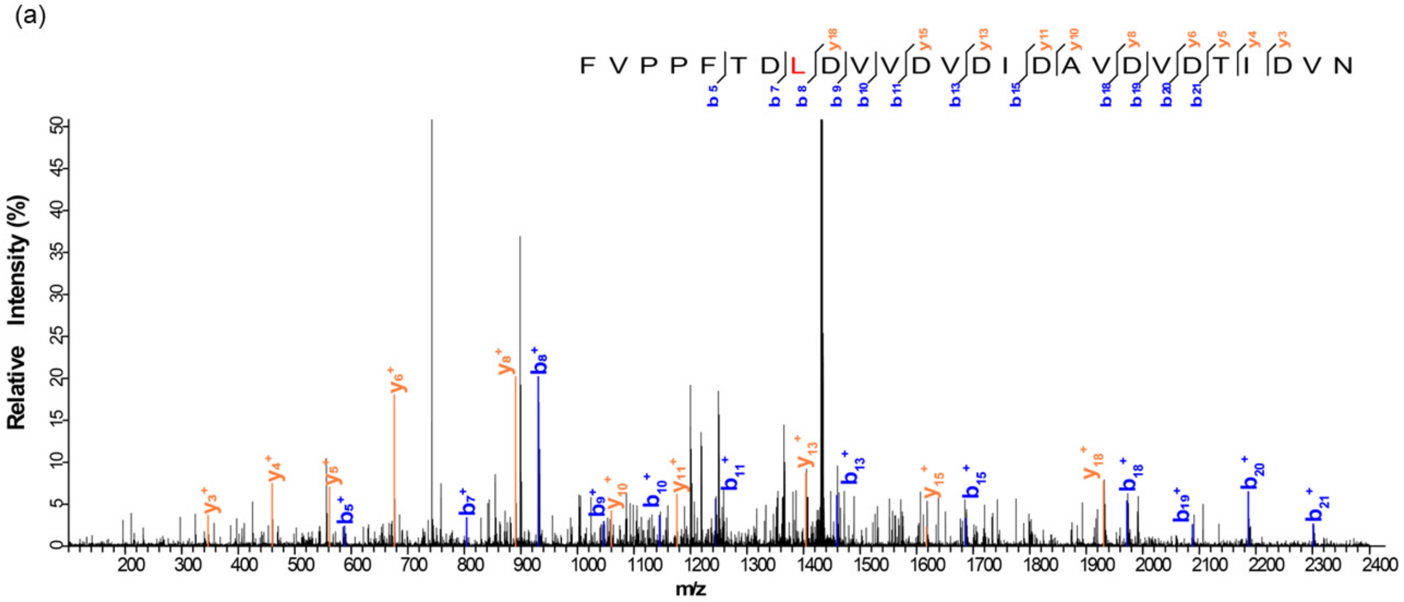

(b)

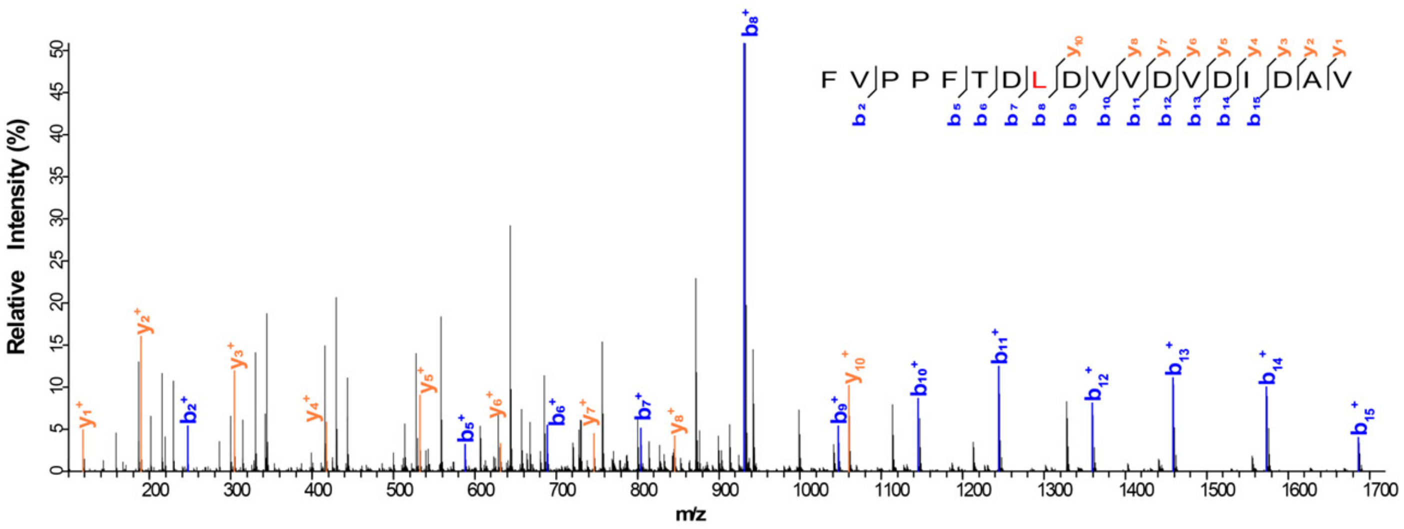
Relative Intensity (%)
m/z
FVPPFTDLDVVDVDIDAV

(c)

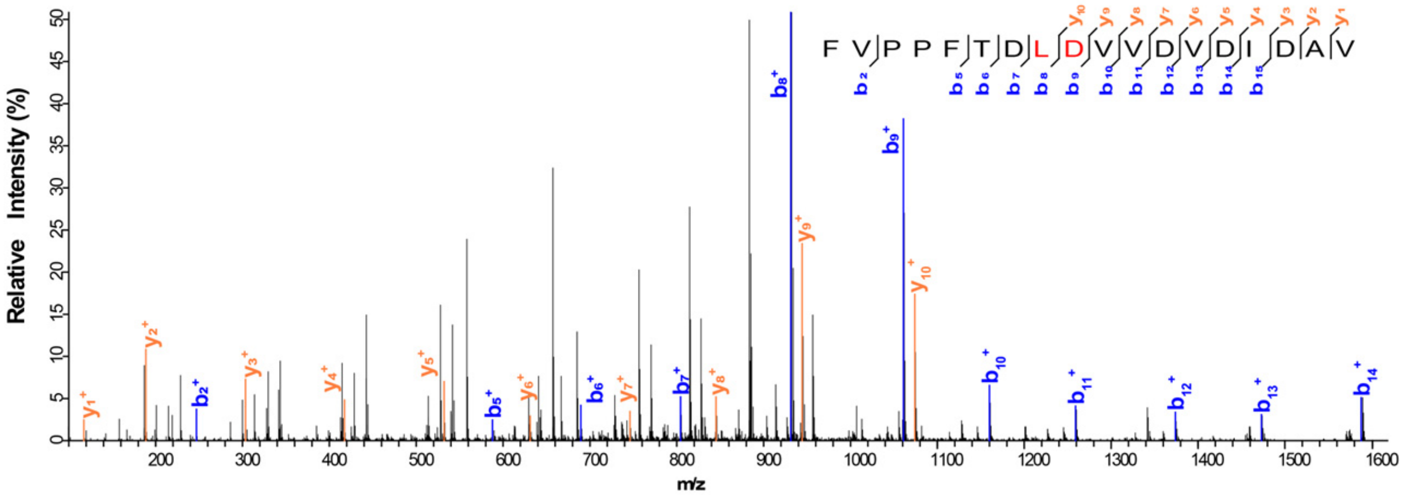
Relative Intensity (%)
m/z
FVPPFTDLDVVDVDIDAV

(d)

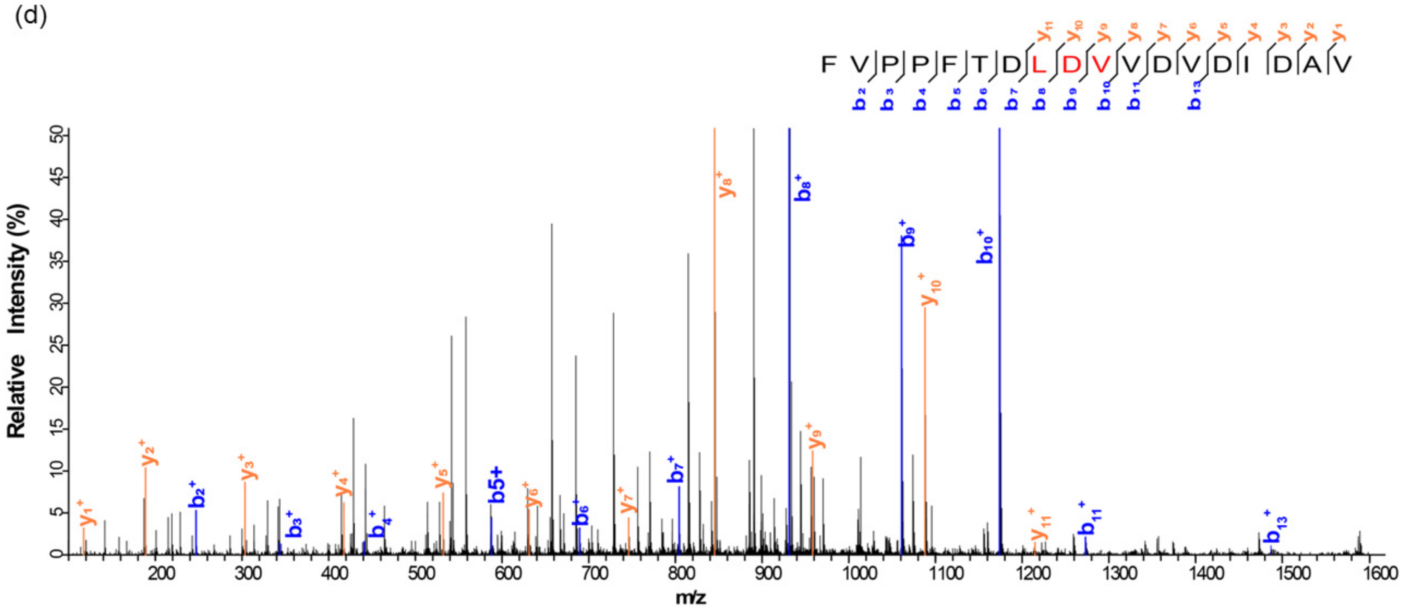

FVPPFTDLDVVDVDIDAV
Relative Intensity (%)
m/z

(e)

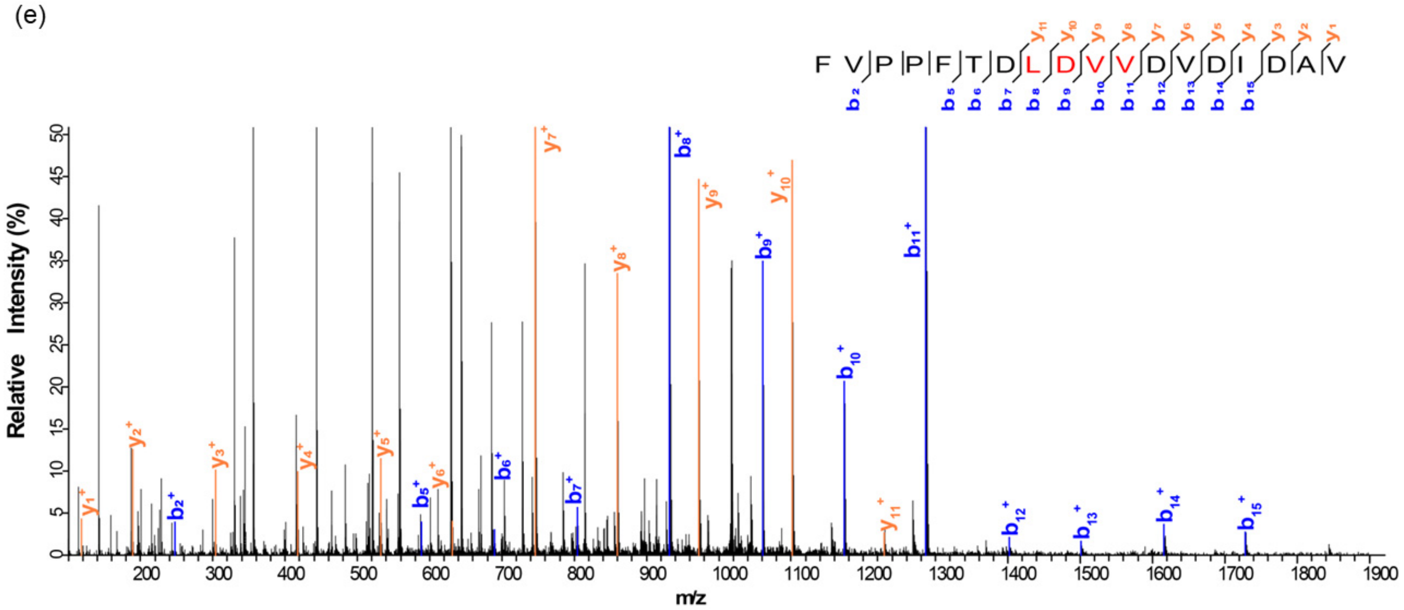

FVPPFTDLDVVDVDIDAV
Relative Intensity (%)
m/z

(f)

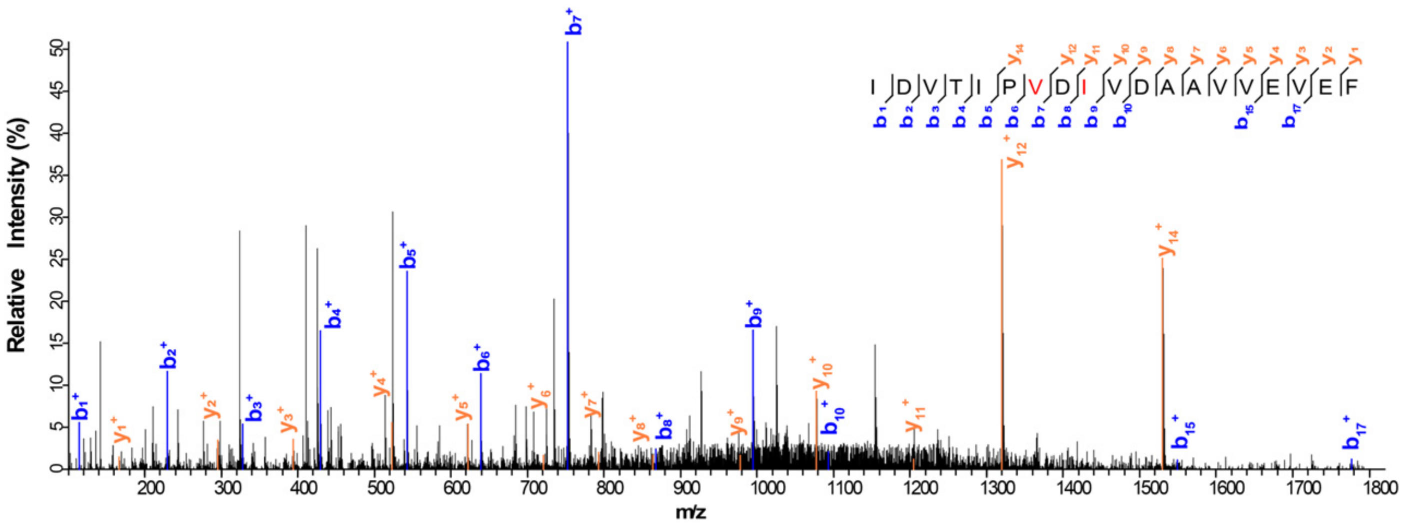

Relative Intensity (%)
m/z
I D V T I P V D I V D A A V V E V E F

(g)

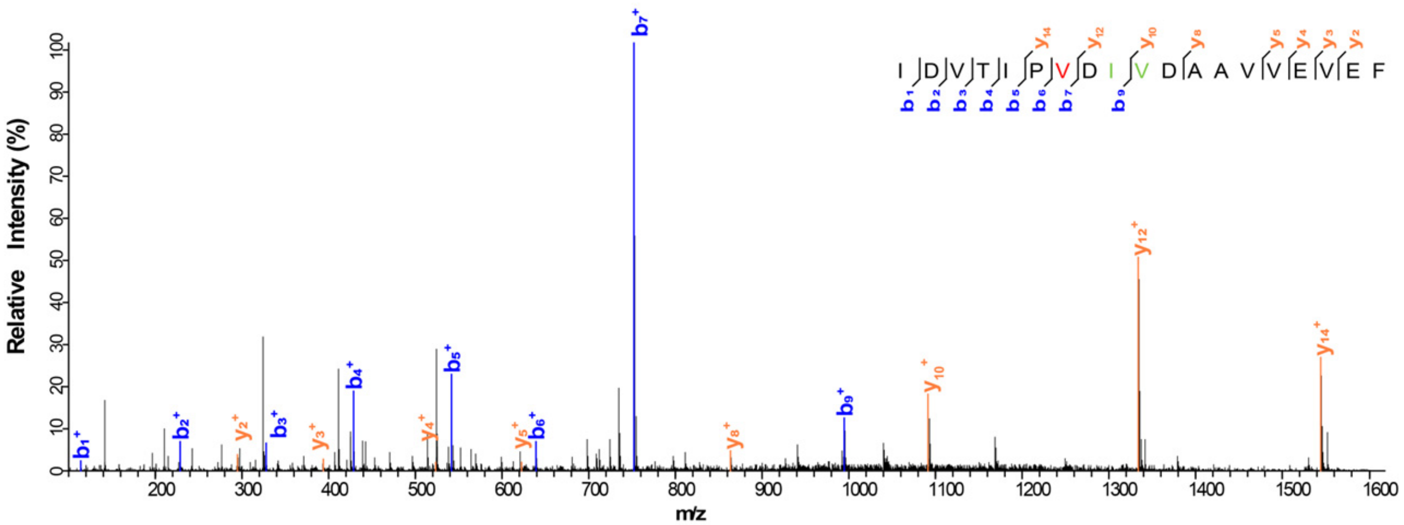

Relative Intensity (%)
m/z
I D V T I P V D I V D A A V V E V E F

(h)

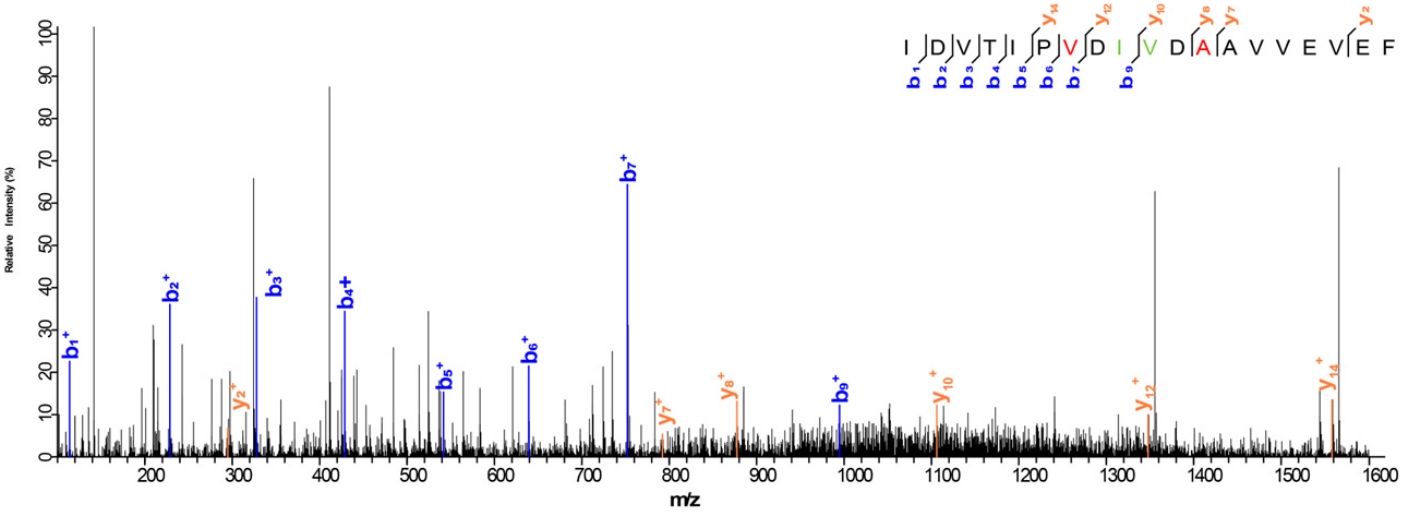

I D V T I P V D I V D A A V V E V E F
Relative Intensity (%)
m/z

(i)

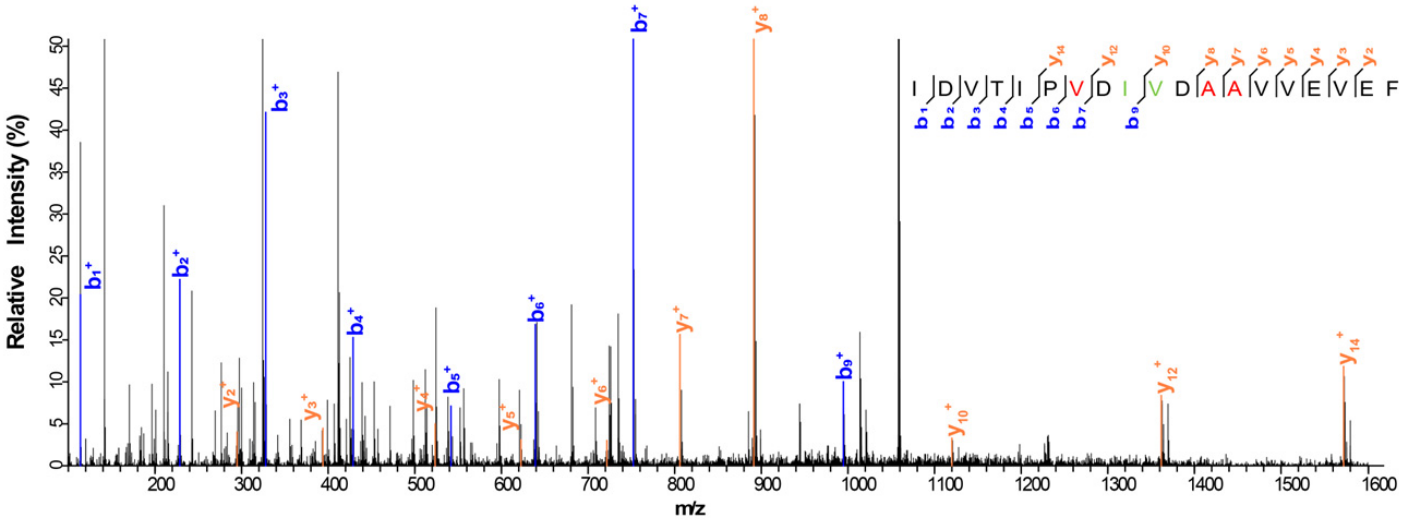

I D V T I P V D I V D A A V V E V E F
Relative Intensity (%)
m/z

(j)

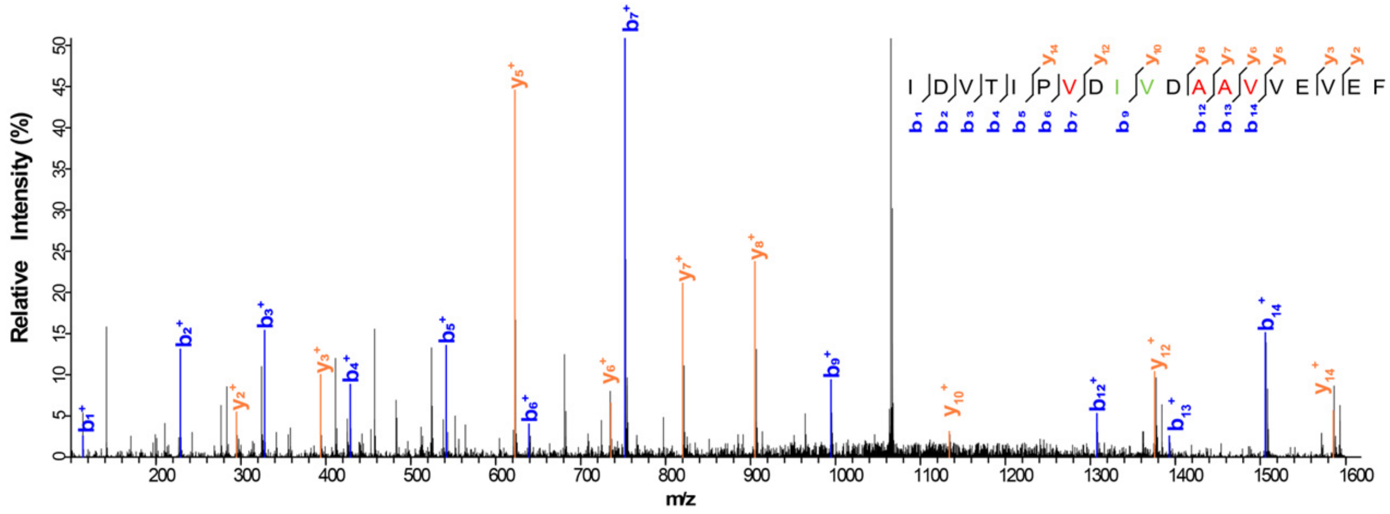

Figure S2. The MS/MS spectra for KchMA reveal methylated residues in proteolytically released core peptide fragments. Methylated amino acids confirmed by MS/MS are represented as red fonts, while methylated amino acids inferred by MS/MS are represented as green fonts.

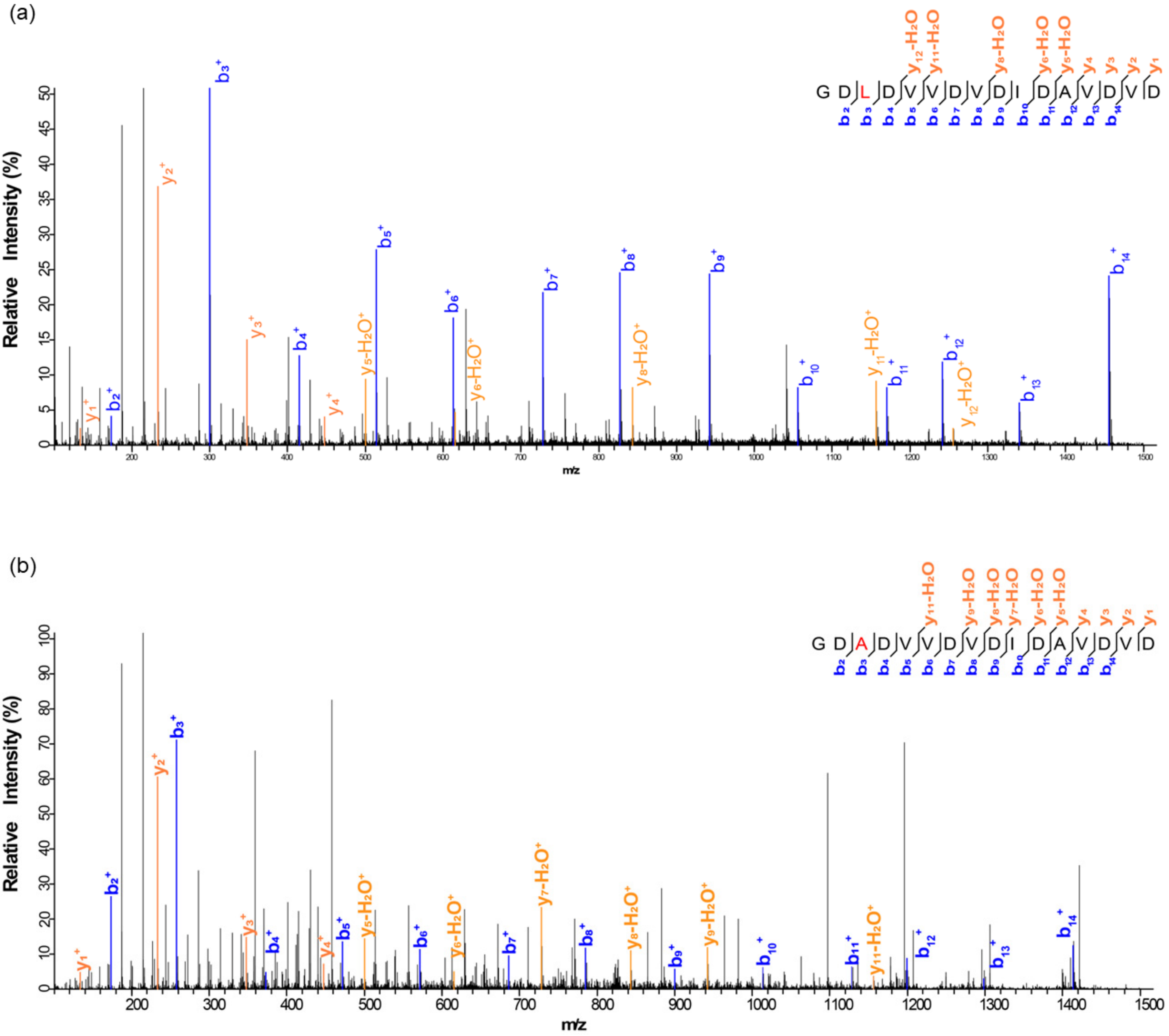
(a)
GDLDVVDVDIDAVDVD
Relative Intensity (%)
m/z
(b)
GDADVVDVDIDAVDVD
Relative Intensity (%)
m/z

(c)

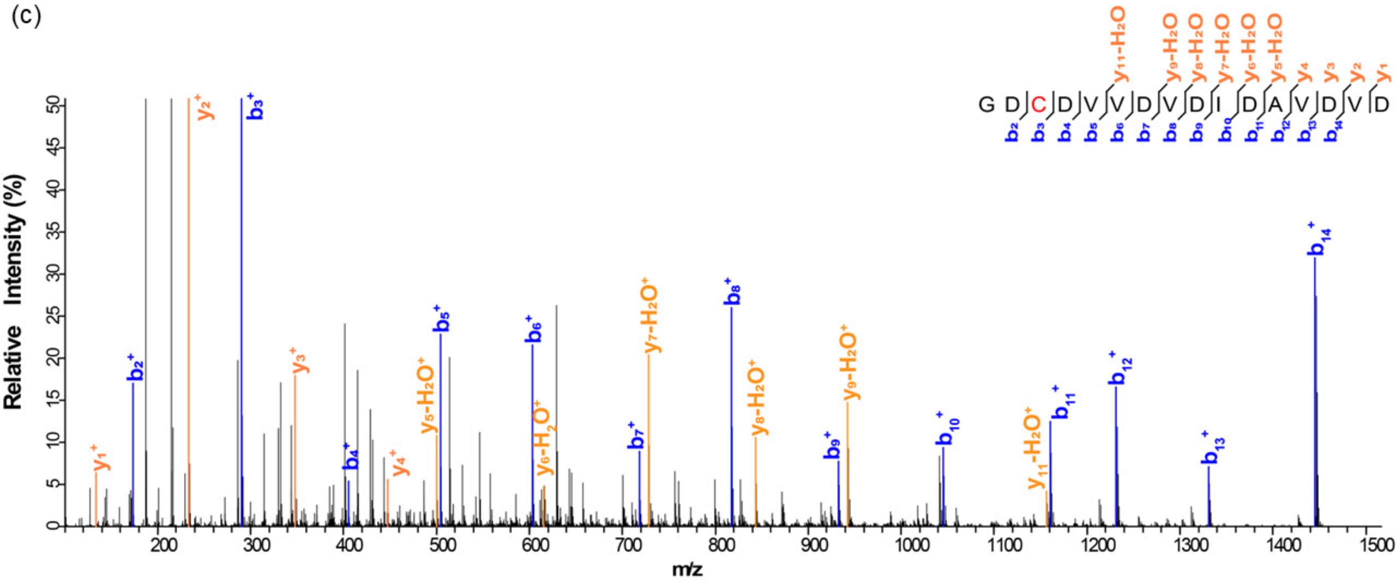

Relative Intensity (%)
m/z
G D C D V V D V D I D A V D V D

(d)

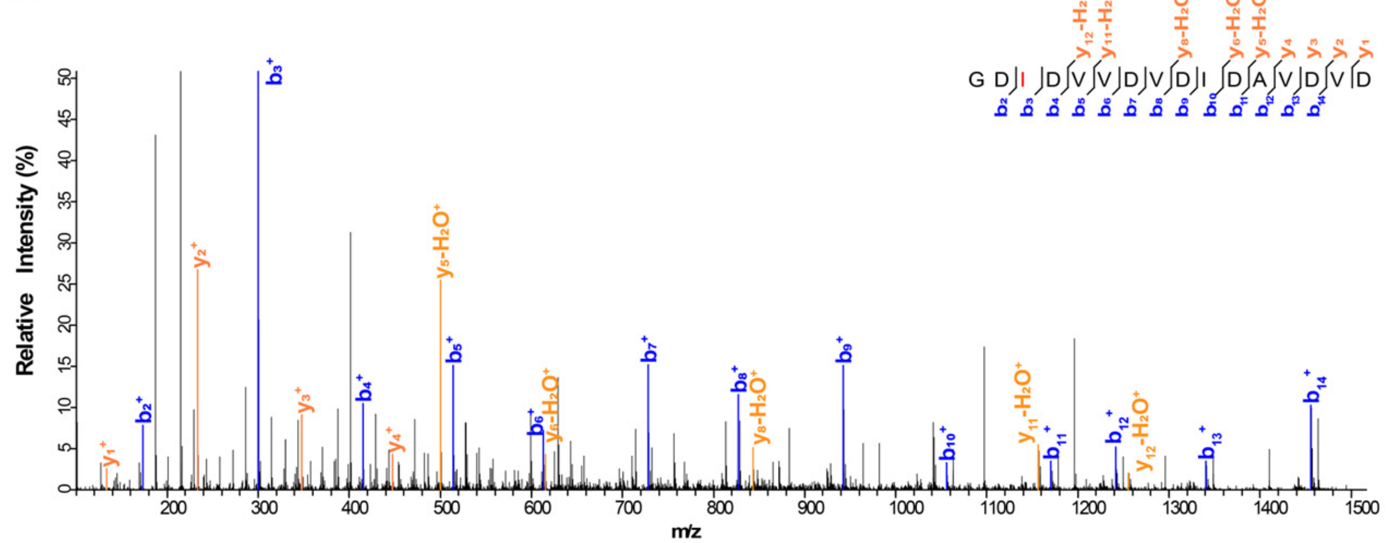

Relative Intensity (%)
m/z
G D I D V V D V D I D A V D V D

(e)

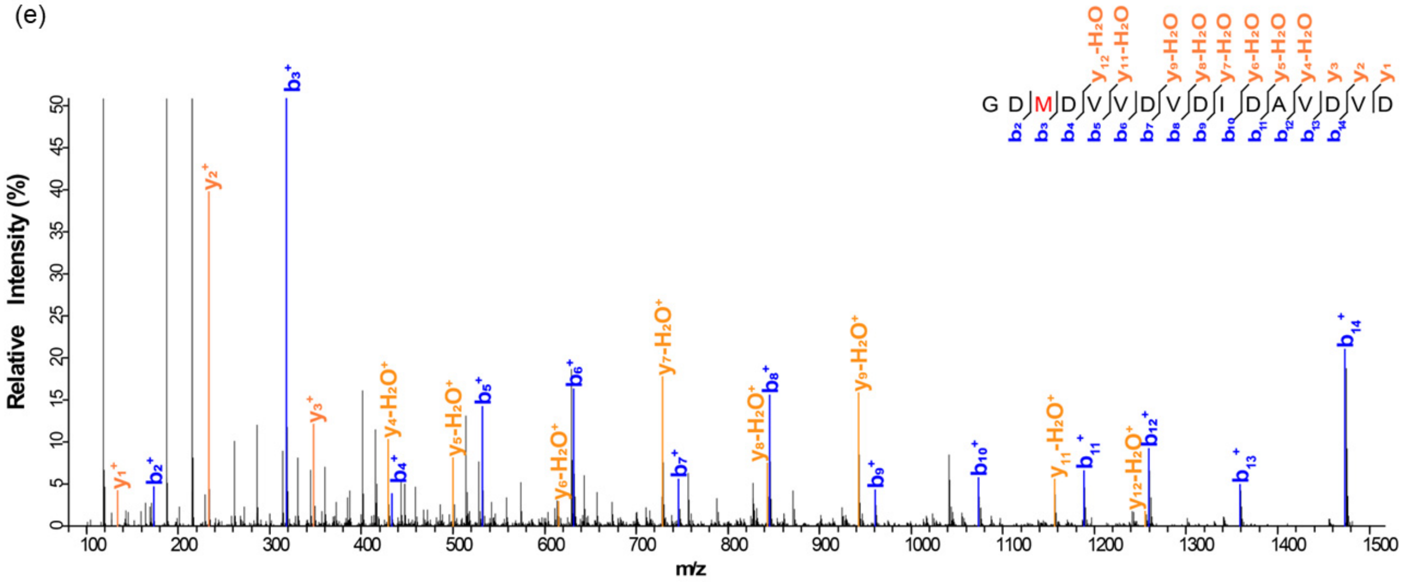
Relative Intensity (%)
m/z
G D M D V V D V D I D A V D V D

(f)

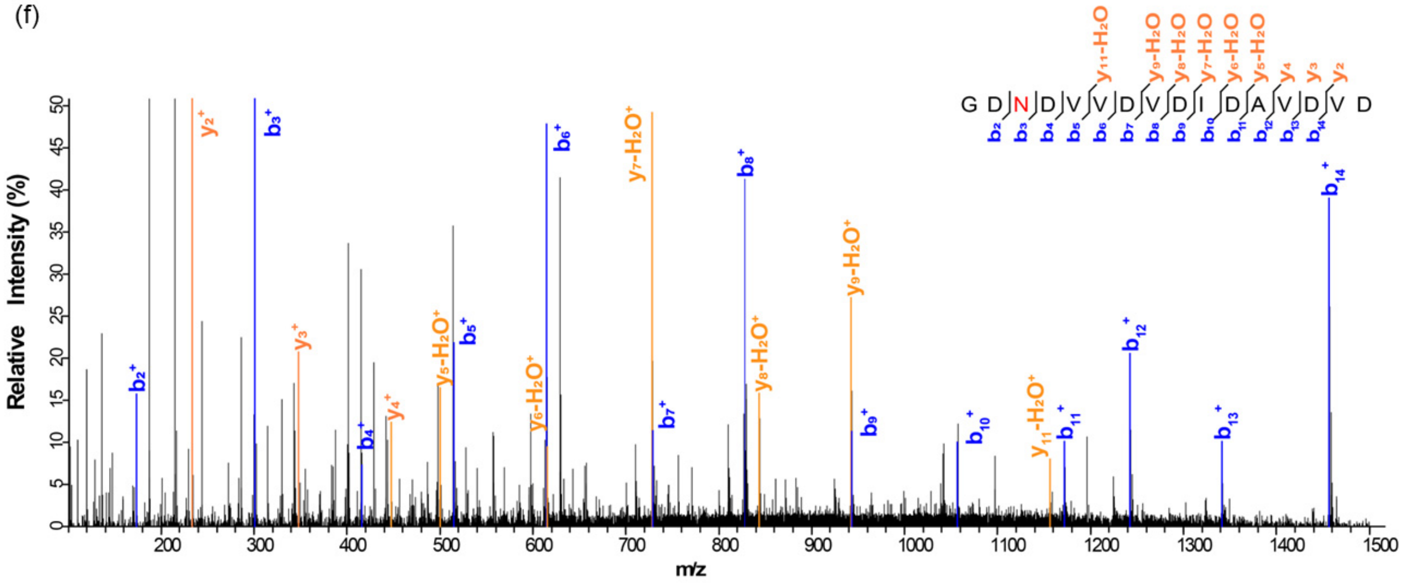
Relative Intensity (%)
m/z
G D N D V V D V D I D A V D V D

(g)

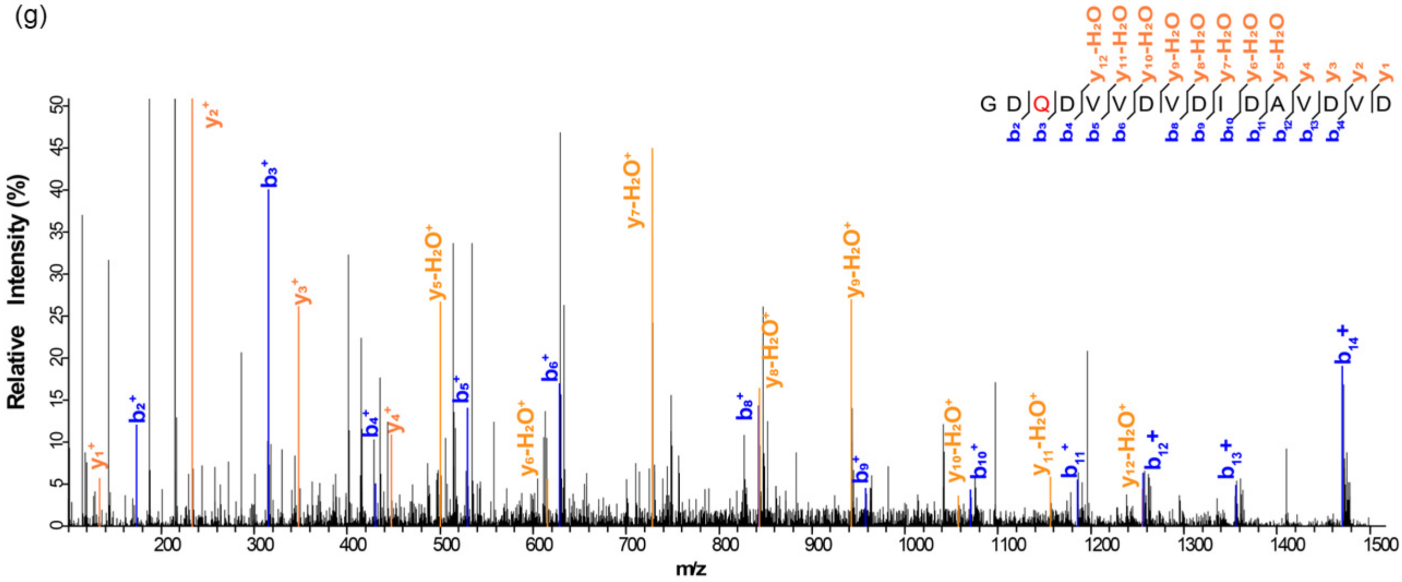
Relative Intensity (%)
m/z
G D Q D V V D V D I D A V D V D
y12-H2O y11-H2O y10-H2O y9-H2O y8-H2O y7-H2O y6-H2O y5-H2O y4 y3 y2 y1
b2 b3 b4 b5 b6 b8 b9 b10 b11 b12 b13 b14
y1+ b2+ y2+ b3+ y3+ b4+ y4+ y5-H2O+ b5+ y6-H2O+ b6+ y7-H2O+ b8+ y8-H2O+ b9+ y9-H2O+ y10-H2O+ b10+ y11-H2O+ b11+ y12-H2O+ b12+ b13+ b14+

(h)

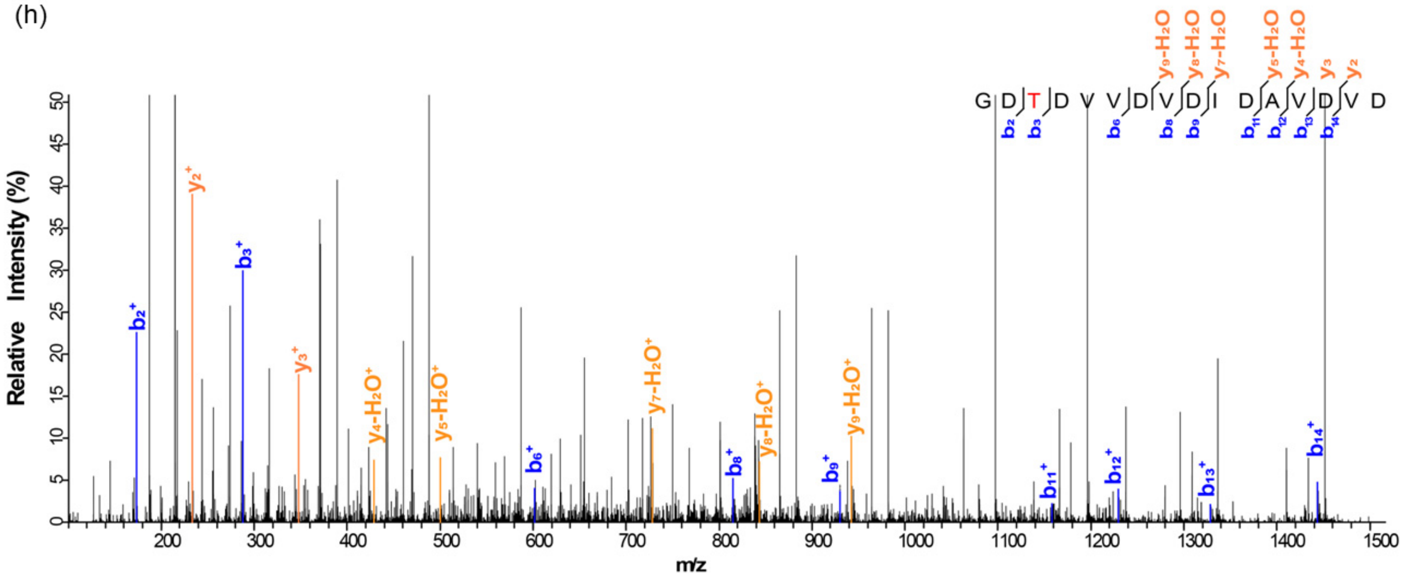
Relative Intensity (%)
m/z
G D T D V V D V D I D A V D V D
y9-H2O y8-H2O y7-H2O y5-H2O y4-H2O y3 y2
b2 b3 b6 b8 b9 b11 b12 b13 b14
b2+ y2+ b3+ y3+ y4-H2O+ y5-H2O+ b6+ y7-H2O+ b8+ y8-H2O+ b9+ y9-H2O+ b11+ b12+ b13+ b14+

(i)

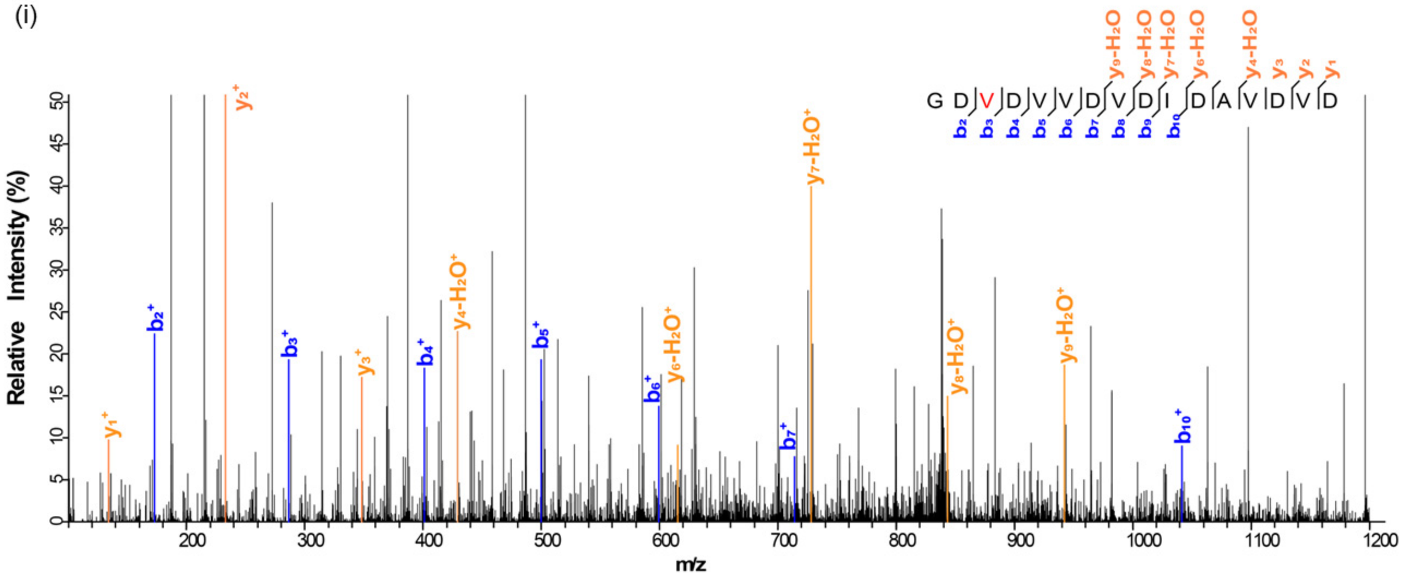

Figure S3. The MS/MS spectra for KchMAtev reveal methylated residues in proteolytically released core peptide fragments. Methylated amino acids confirmed by MS/MS are represented as red fonts.

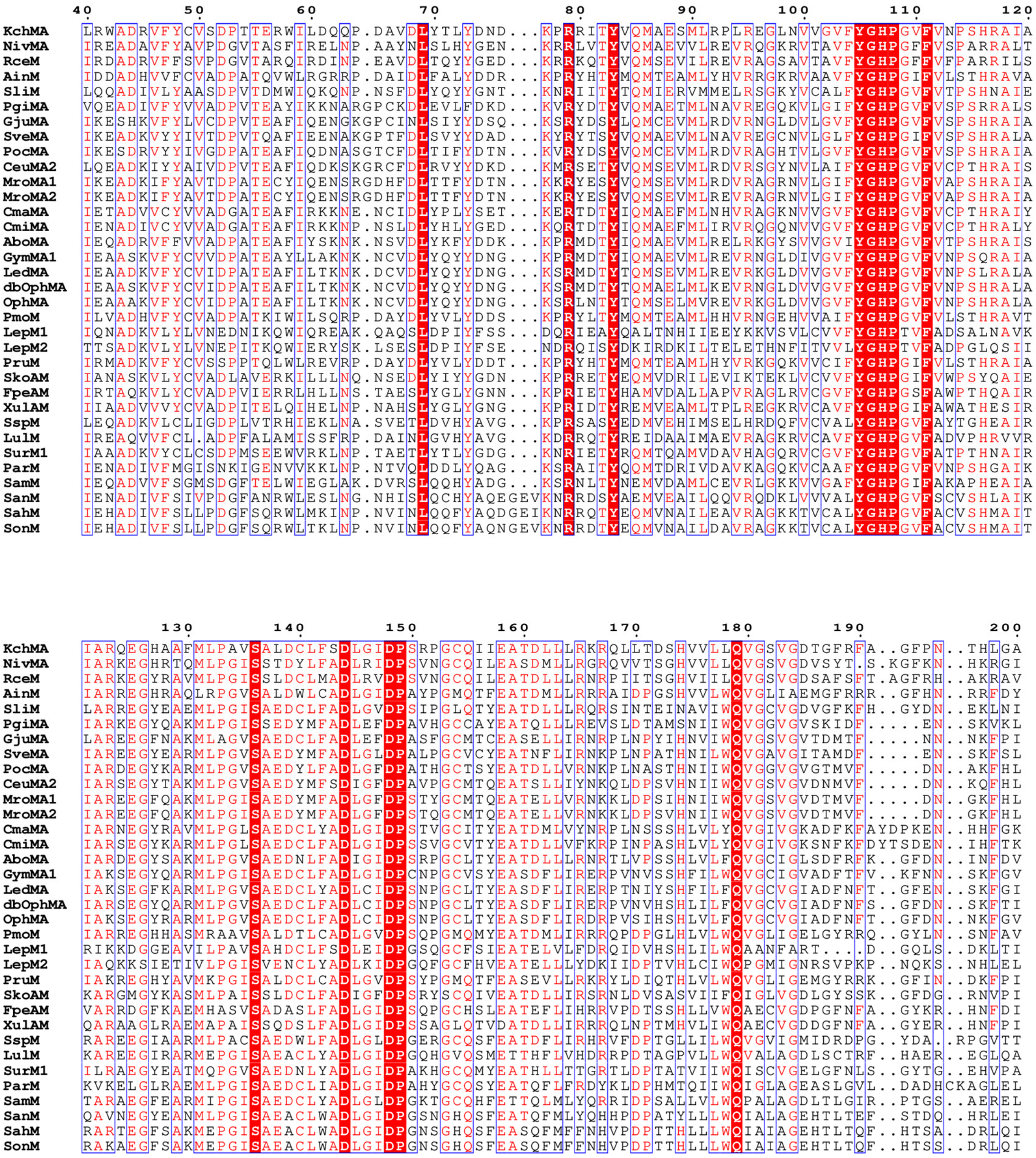

Figure S4. Sequence alignment of characterized borosins. A percentage of conserved residues is calculated per columns. Highly conserved residues and motifs are colored in red and framed in blue.

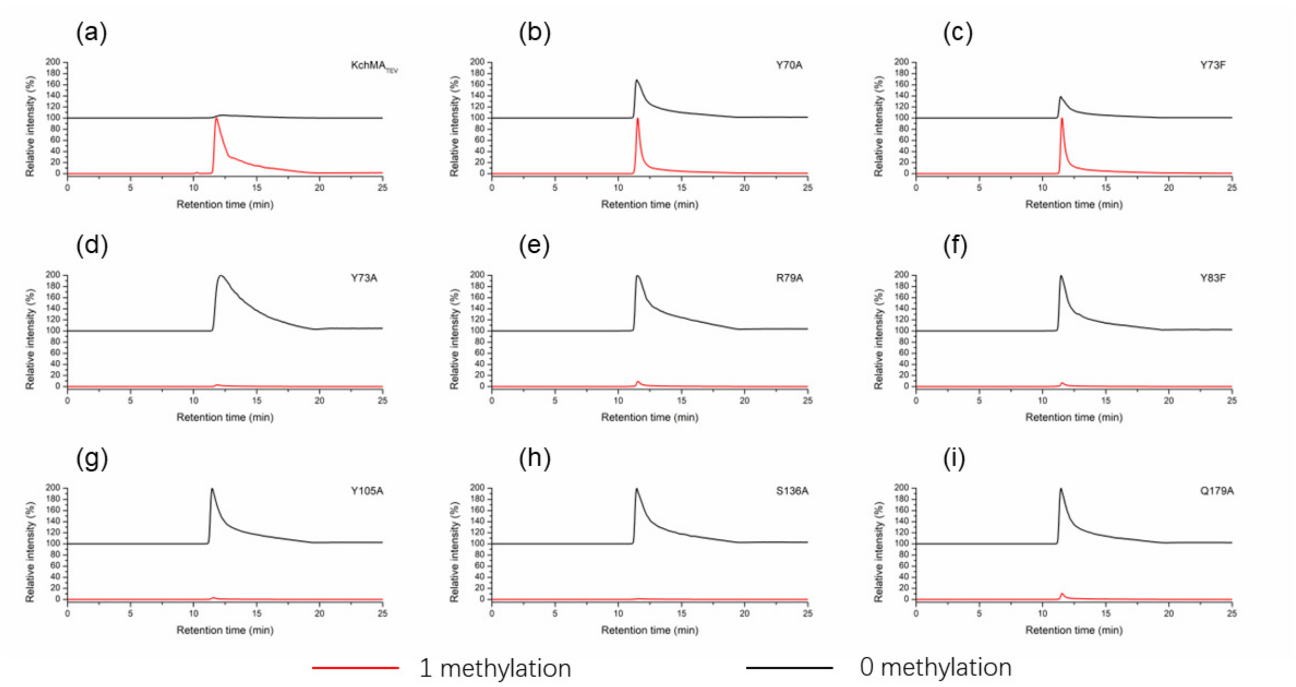

Figure S5. Identification of the active sites in KchMA$_{TEV}$.

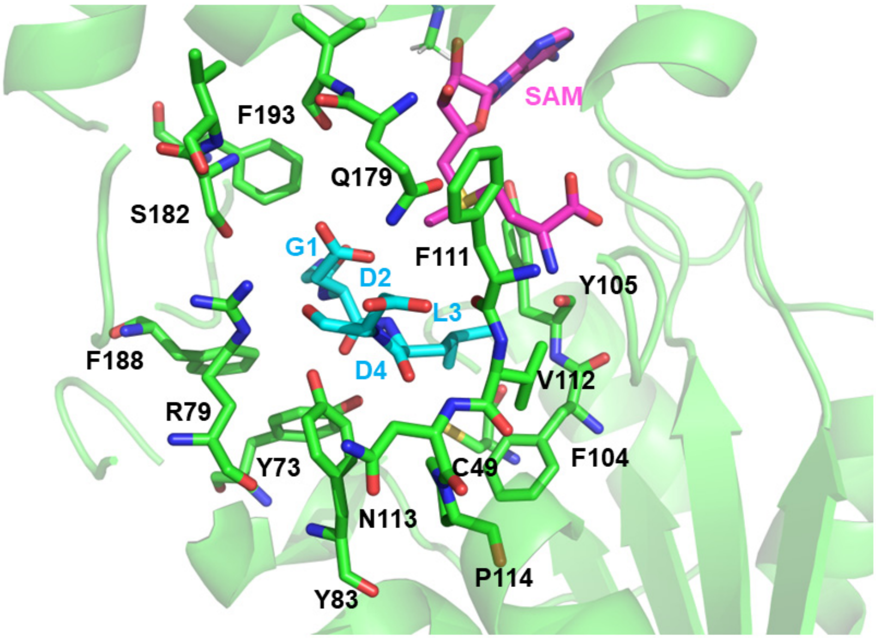

Figure S6. The residues close to D2 and L3 of core peptide within 5 Å. The fragment of the core peptide is colored in cyan, while the residues of the methyltransferase domain are colored in green. SAM is colored in pink.

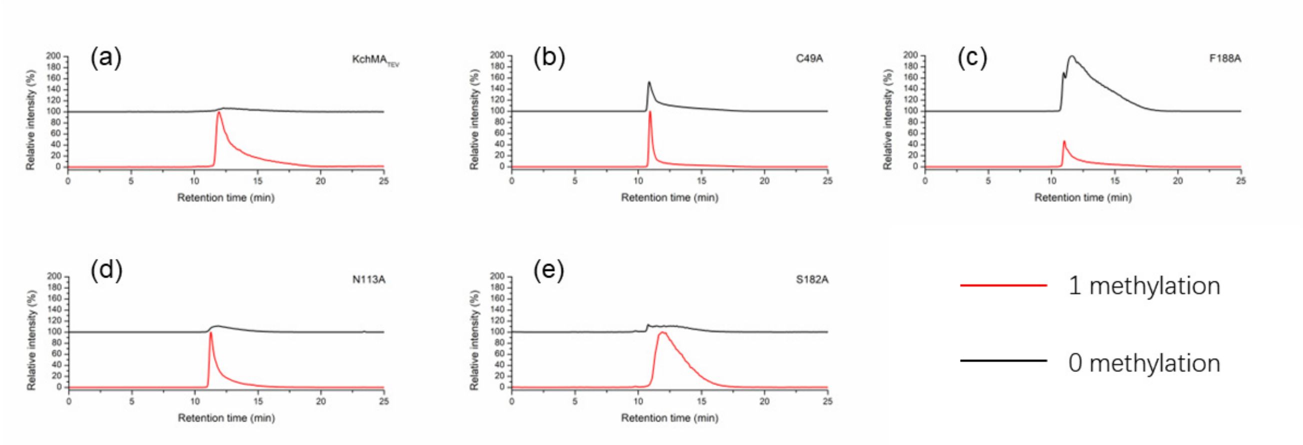

Figure S7. Alanine scan experiments targeting sites within the active site that are lower conserved. C49A and F188A showed a significant decrease in methylation activity.

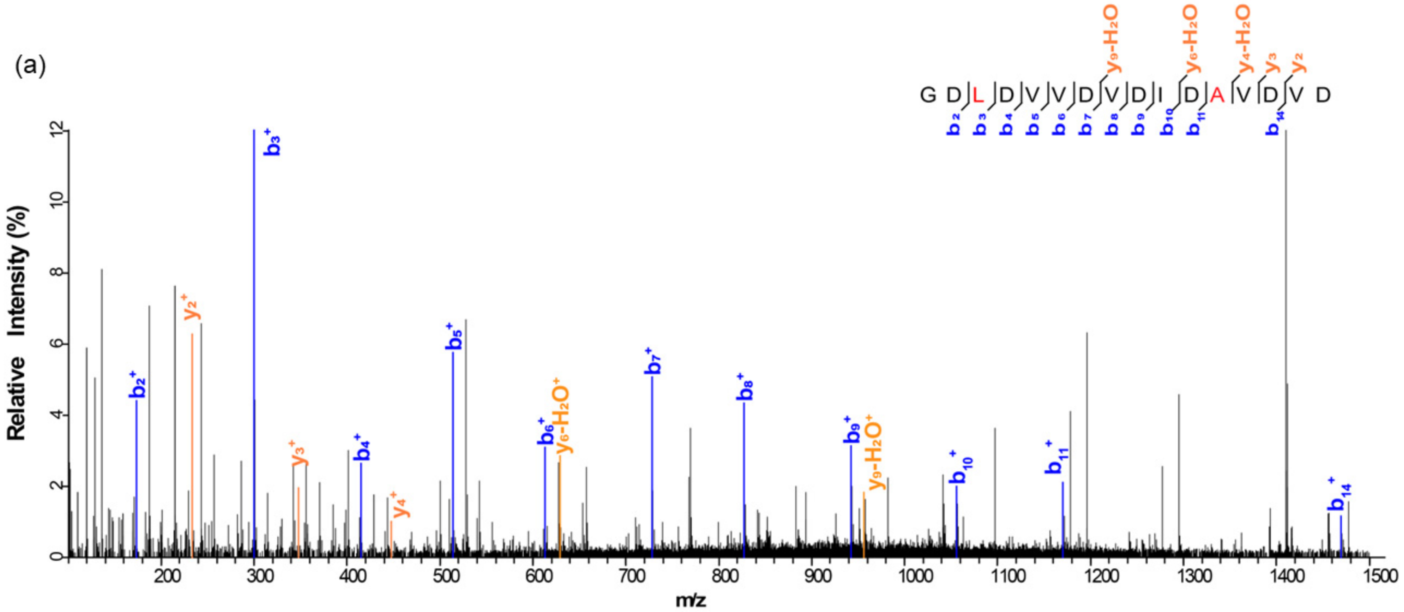

(b)

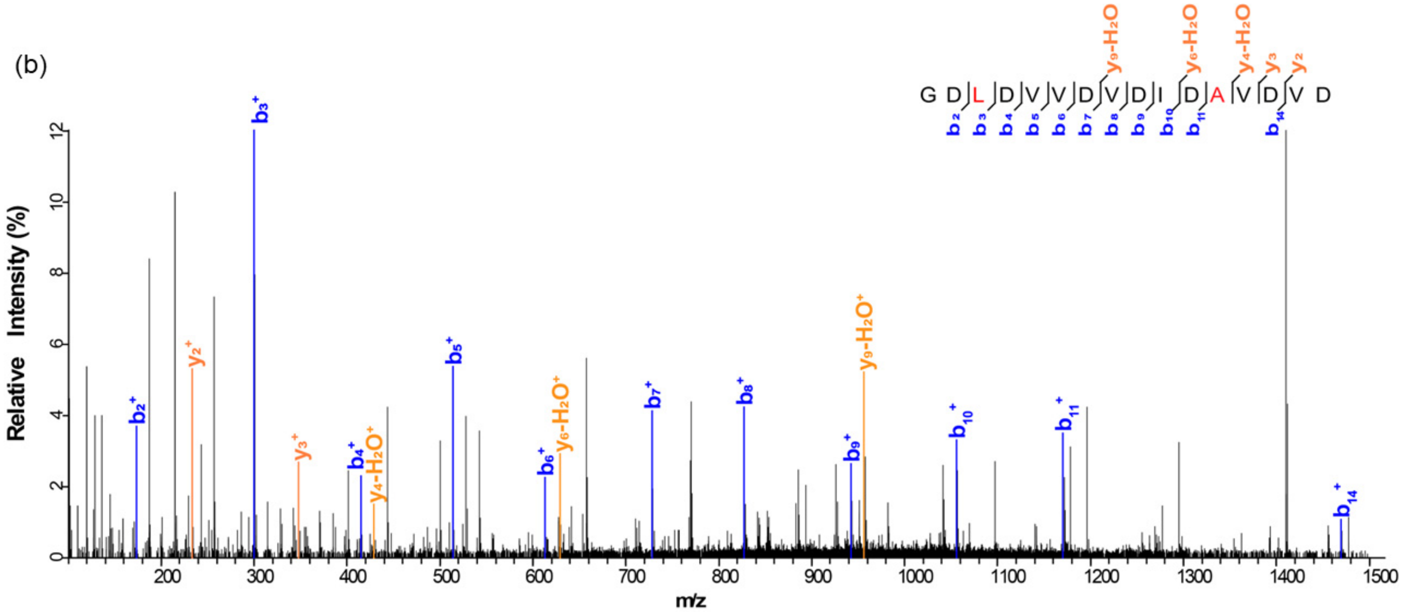
G D L D V V D V D I D A V D V D
Relative Intensity (%)
m/z
b2+
y2+
b3+
y3+
b4+
y4-H2O+
b5+
b6+
y6-H2O+
b7+
b8+
b9+
y9-H2O+
b10+
b11+
b14+

(c)

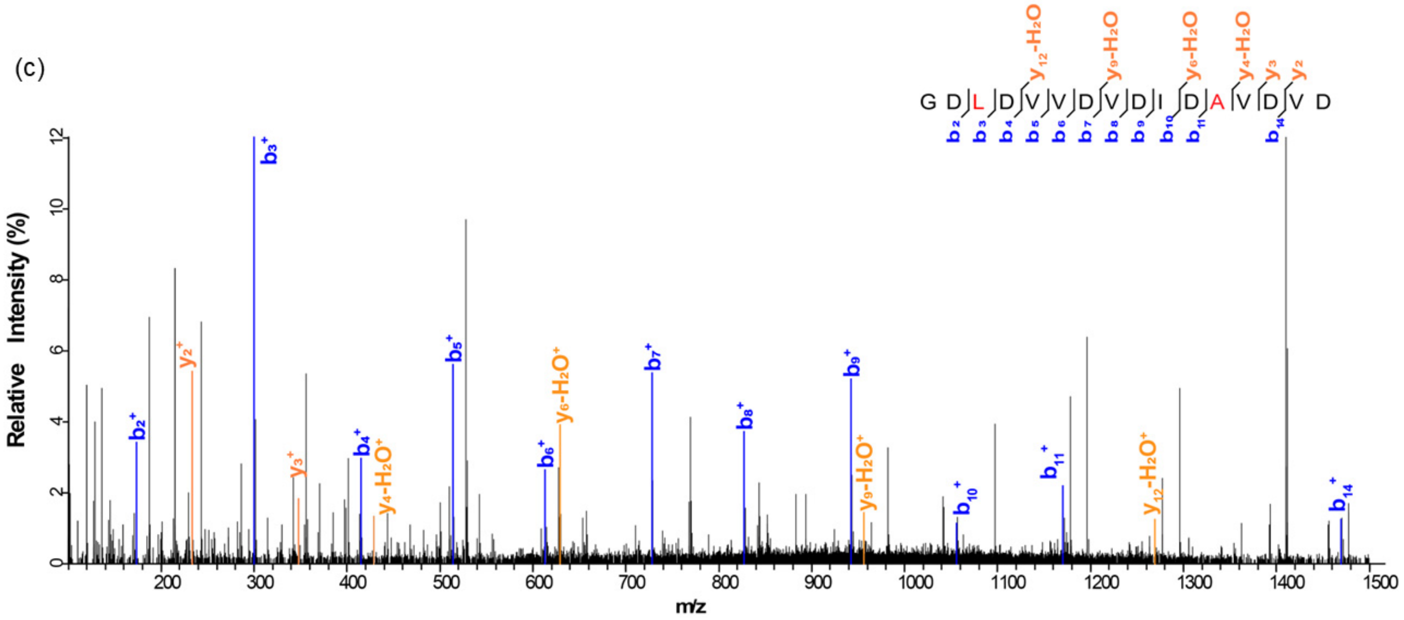
G D L D V V D V D I D A V D V D
Relative Intensity (%)
m/z
b2+
y2+
b3+
y3+
b4+
y4-H2O+
b5+
b6+
y6-H2O+
b7+
b8+
b9+
y9-H2O+
b10+
b11+
y12-H2O+
b14+

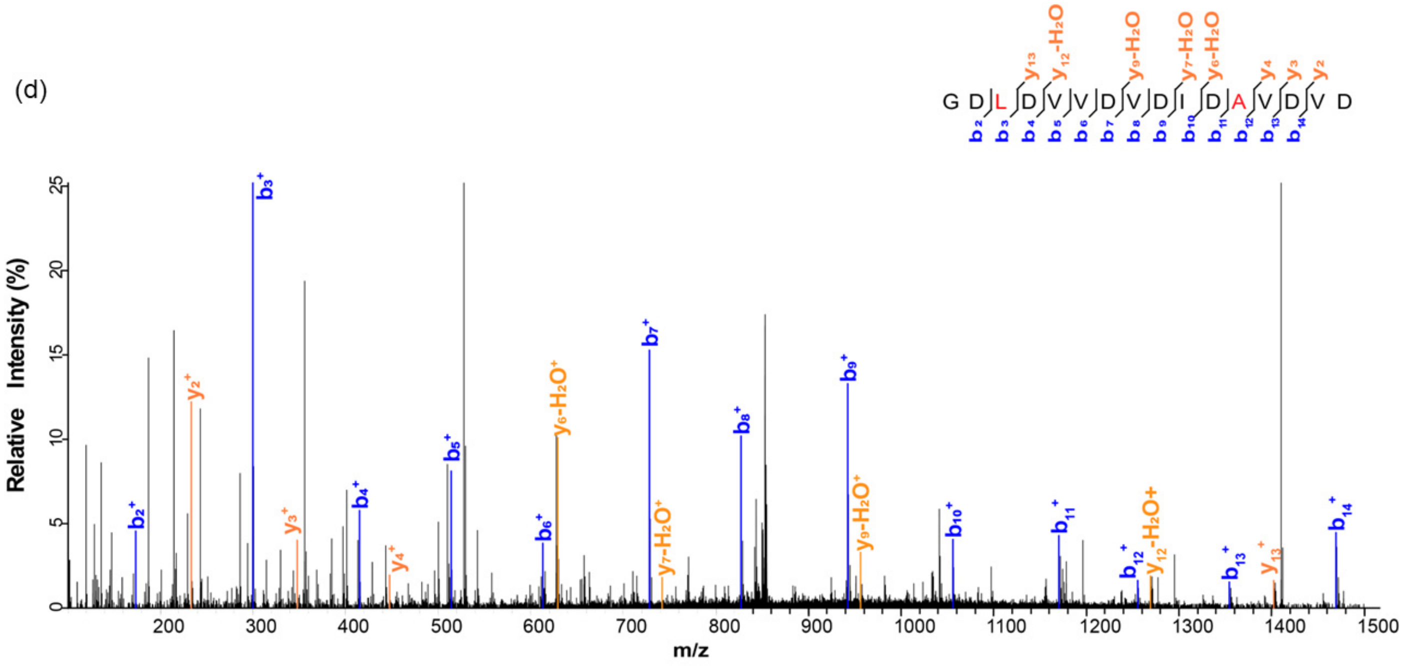

Figure S8. The MS/MS results for dimethylation product of (a) M1, (b) M2, (c) M4 and (d) M7.
